# The Combination of Nirmatrelvir and Ivermectin Exerts Strongly Synergistic Antiviral and Anti-Inflammatory Effects Against Murine Coronavirus Infection of Macrophages

**DOI:** 10.3390/ijms27167316

**Published:** 2026-08-16

**Authors:** Wilson Z. Y. How, Le Xin Teh, Vincent T. K. Chow

**Affiliations:** 1 Department of Microbiology and Immunology, Yong Loo Lin School of Medicine, National University of Singapore, Kent Ridge, Singapore 117545, Singapore; wilsonhzy@gmail.com (W.Z.Y.H.); lexin.teh02@gmail.com (L.X.T.); 2 Infectious Diseases Translational Research Program, Yong Loo Lin School of Medicine, National University of Singapore, Kent Ridge, Singapore 117545, Singapore

**Keywords:** Nirmatrelvir, Ivermectin, Azithromycin, Doxycycline, antiviral, anti-inflammatory, combination therapy, repurposed drugs, murine hepatitis virus, coronavirus

## Abstract

The emergence of SARS-CoV-2 variants and antiviral resistance highlights the need for improved therapeutic strategies against COVID-19 and other coronavirus infections. By pairing direct-acting antivirals with repurposed host-modulating drugs, combination therapy approaches may enhance antiviral efficacy while mitigating inflammation. In this study, we evaluated the effects of combining Nirmatrelvir (a SARS-CoV-2 main protease inhibitor) with Ivermectin, Azithromycin or Doxycycline—using a murine hepatitis virus (MHV) infection model of RAW264.7 macrophages. Checkerboard assays demonstrated that the Nirmatrelvir–Ivermectin combination exhibited strongly synergistic antiviral activity. This combination achieved potent inhibition of live virus titer of at least 5 to 6 log_10_ and reduction in viral RNA load of 3 log_10_, at drug concentrations much lower than the respective monotherapies. The Nirmatrelvir–Ivermectin combination treatment affected the coronavirus replication cycle at the same time-point of 8 h as Nirmatrelvir monotherapy. Moreover, multiplex cytokine protein profiling revealed that Nirmatrelvir–Ivermectin markedly suppressed key pro-inflammatory cytokines and chemokines associated with the cytokine storm, including IL-6, TNF-α, IL-1β, and MCP-1. Conversely, the combinations of Nirmatrelvir with Azithromycin or Doxycycline exhibited only additive or mildly additive effects, with alterations in certain cytokine levels. These findings support the potential of Nirmatrelvir–Ivermectin as a promising novel combination therapy with both antiviral and anti-inflammatory benefits, warranting further in vivo validation and clinical investigation.

## 1. Introduction

The severe acute respiratory syndrome coronavirus 2 (SARS-CoV-2) has infected about 780 million people globally to date, and led to a worldwide shutdown culminating in huge socioeconomic losses during the COVID-19 pandemic. SARS-CoV-2 continues to evolve with multiple mutations resulting in the emergence of new viral variants associated with varying disease severity. Despite the widespread availability of vaccines, these are unable to provide complete protection against COVID-19, thus necessitating efficacious drug treatments [1,2,3]. The pandemic prompted the rapid development of therapeutics against SARS-CoV-2, with many pharmaceutical companies investing in the discovery of effective treatment options such as antiviral drugs [4,5]. These include Remdesivir, Paxlovid, and Molnupiravir, which were approved for emergency clinical use against COVID-19. Despite their demonstrated antiviral efficacy, there remain certain limitations and challenges to resolve. Some limitations include their adverse side-effects, narrow therapeutic window, and diminished efficacy against rapidly mutating SARS-CoV-2 variants due to drug resistance. For example, the Ritonavir component in Paxlovid is a very strong inhibitor of the CYP3A cytochrome P450 enzyme group, and may cause drug–drug interactions by dangerously raising the levels of other medications such as statins and anticoagulants [6,7,8,9,10].

Remdesivir is a viral RNA polymerase inhibitor that requires intravenous administration and incurs high treatment costs of approximately US$3000 for a standard 5-day course, thus limiting its accessibility in low- and middle-income regions [11]. Furthermore, one study showed that SARS-CoV-2 is capable of acquiring resistance to Remdesivir, although this was observed in vitro and may not reflect the actual complexities of in vivo systems [12,13]. Nirmatrelvir is a 3CL protease inhibitor co-administered with Ritonavir (in the form of oral Paxlovid), and has shown reduced efficacy in certain emerging drug-resistant SARS-CoV-2 strains [14].

One viable strategy to overcome these challenges and limitations is by combination therapy with repurposed drugs. There are multiple studies which prove that combining therapeutics is effective, with successful examples in infectious diseases such as tuberculosis. Thus, the repurposed drugs Moxifloxacin, Linezolid, and Clofazimine are harnessed to treat extensively drug-resistant tuberculosis when conventional antimicrobial regimens fail. Additionally, for HIV treatment, combining antiviral treatment with drugs which possess complementary mechanisms can enhance treatment efficacy while minimizing resistance. These precedents suggest that combining antiviral agents with host-directed or repurposed drugs may similarly improve outcomes in COVID-19 management [15,16,17,18].

The central hypothesis underlying this approach is that combinations of clinically approved drugs which target both viral and host pathways, may produce synergistic effects, leading to greater antiviral potency and lower toxicity compared to monotherapy [19,20]. The combination of Remdesivir and Ivermectin can exert strongly synergistic effects against coronaviruses compared to the monotherapies [20,21]. Combination therapy has the benefit of reducing the emergence of escape mutants, while improving the safety profile of treatment, since lower amounts of each constituent drug are needed [22]. Repurposed drugs such as Ivermectin [23,24], Doxycycline [25,26], and Azithromycin [27,28] are purported to exert antiviral and/or immunomodulatory effects. Ivermectin interferes with importin-mediated nuclear transport, and may suppress STAT3-mediated IL-6 expression [29]. Doxycycline inhibits metalloproteinases (MMPs), and downregulates cytokines [30]. Azithromycin affects host-virus binding and modulates NF-κB pathways [31].

The objective of this study was to identify combination therapies with potential pan-coronavirus inhibitory activity by targeting SARS-CoV-2 and other coronaviruses. Our study investigated the combinations of Nirmatrelvir (an approved oral antiviral agent against SARS-CoV-2) paired with three repurposed drugs (Ivermectin, Azithromycin, and Doxycycline) against infection of the RAW264.7 macrophage cell line with murine hepatitis virus (MHV), a representative betacoronavirus model. Monocytes and macrophages are primary innate immune cells first encountered by coronaviruses, and constitute the main producers of inflammatory cytokines and chemokines in response to the infection [32,33]. In addition to the known virus-targeting effects of current antivirals, the repurposed drugs were hypothesized to augment the former by exerting host-targeting antiviral and immunomodulating effects. Hence, the antiviral and immunomodulatory effects of these drug combinations were assessed and compared. The combination of Nirmatrelvir and Ivermectin was found to possess highly synergistic antiviral and anti-inflammatory effects against MHV infection of macrophages.

## 2. Results

### 2.1. Nirmatrelvir Monotherapy Inhibits MHV Replication in RAW264.7 Cells with Minimal Toxicity

**Figure 1:**
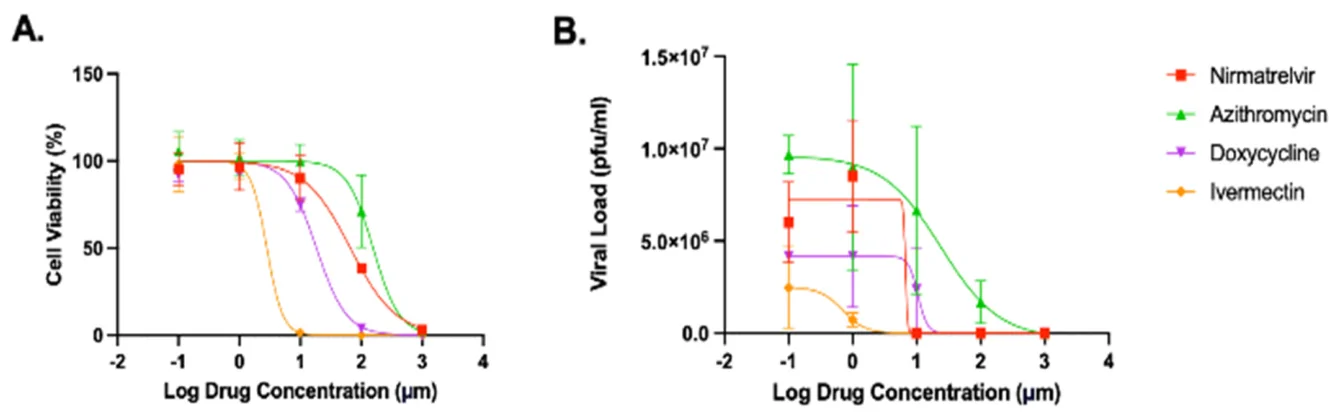
Dose–response curves of individual drugs on cell viability of RAW264.7 cells and inhibition of live MHV. (**A**) Cytotoxic profiles of drugs on RAW264.7 cells were evaluated by the MTS assay (*n* = 3). (**B**) Inhibitory effects of drugs against MHV infection of RAW264.7 cells were quantified by the virus plaque assay (*n* = 3). The standard error of the mean (SEM) of three biological replicates is represented by an error bar. The drug concentration of zero µM refers to the negative control without any drug treatment.

The safety and efficacy profiles of the individual drugs were determined. To assess the safety profiles of the individual drugs, cell viability assays were conducted on RAW264.7 cells, yielding dose–response curves reflecting cellular viability in response to drug treatment (Figure 1A). The CC50 values for each drug were also determined (Table 1). Among the repurposed drugs, Ivermectin demonstrated the lowest CC50 value, indicating relatively higher cytotoxicity.

Next, we evaluated and compared the efficacy of the individual drugs in inhibiting live viral load in MHV-infected RAW264.7 cells (Figure 1B). Nirmatrelvir and Ivermectin demonstrated IC50 values at relatively low concentrations of 6.68 μM and 0.70 μM, respectively (Table 1).

Selectivity indices were calculated to identify drugs that may pose a greater risk of toxicity to host cells than efficacy against the virus. The FDA-approved antiviral Nirmatrelvir exhibited the highest selectivity index compared to the repurposed drugs (Table 1). With a selectivity index of almost 10, Nirmatrelvir effectively inhibited MHV replication with minimal cytotoxicity, indicating a substantial safety margin by being more selective in exerting its therapeutic effect while minimizing cytotoxicity to host cells. Among the repurposed drugs, Azithromycin displayed the highest selectivity index of 7.17 (Table 1).

Based on the calculated CC50 and IC50 values, the concentrations of each drug were selected for evaluation in the checkerboard assays which aimed to explore the efficacy of each combination therapy.

### 2.2. Combination Therapy of Nirmatrelvir and Ivermectin Exhibits Synergistic Effects on Virus Inhibition of MHV

**Figure 2:**
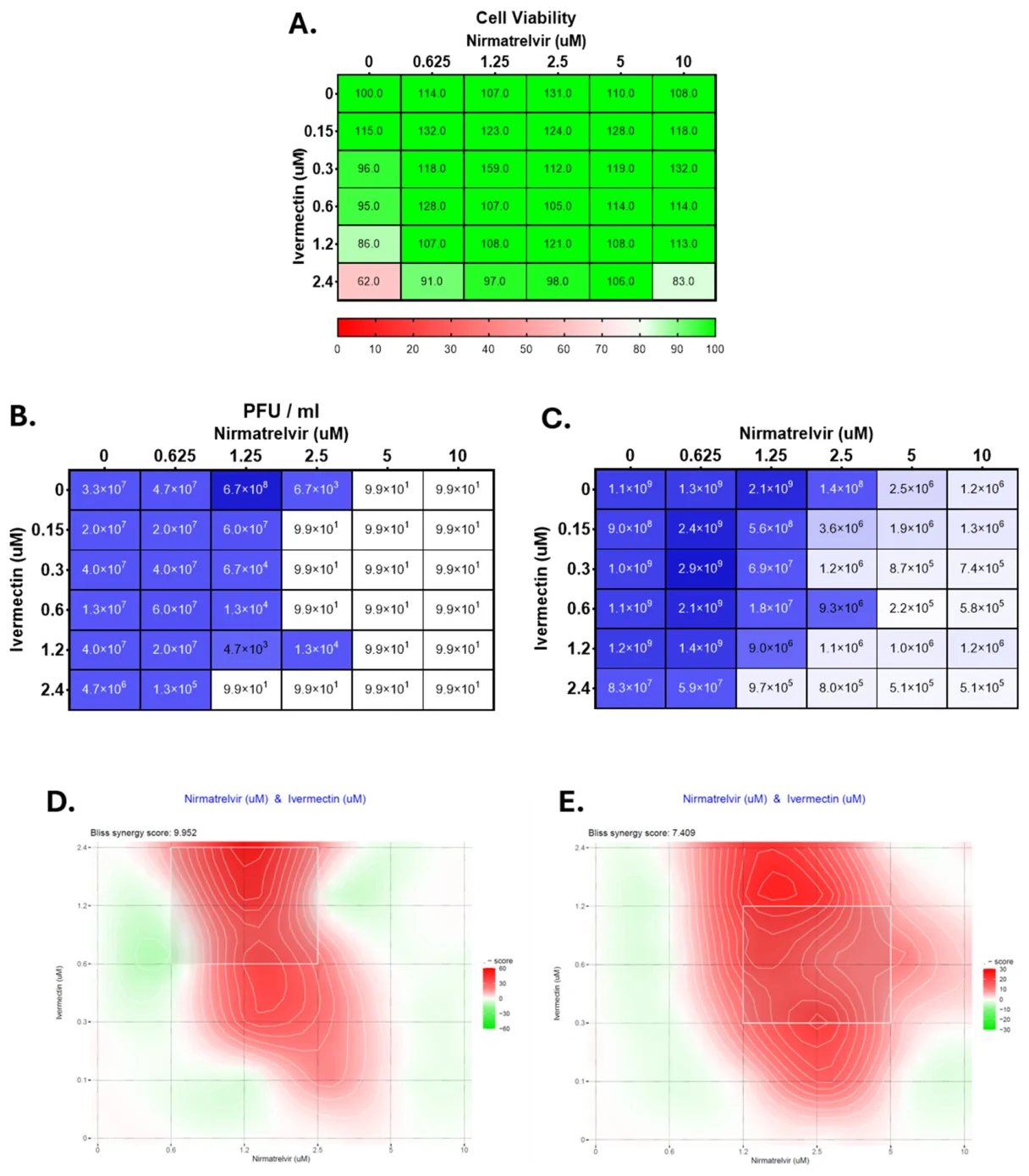
Checkerboard assays of the Nirmatrelvir and Ivermectin combination treatment of RAW264.7 cells. (**A**) Cytotoxicity profile of the drug combination on RAW264.7 cells as measured by the MTS assay. The reference control was healthy cells (without infection and without drug treatment) with an assigned cell viability of 100%. Hence, cell viability percentages above 100% reflect viable healthy cells. Cell viability of 80% or greater is generally accepted for a drug treatment to be considered as non-cytotoxic. (**B**) Live viral titer of MHV following specified drug treatment of MHV-infected RAW264.7 cells as quantified by virus plaque assay. The value of 9.9 × 10^1^ (=99) indicates a live virus titer of less than 100 PFU/mL. The darker shades of blue denote higher viral loads, whereas the white areas reflect very low viral loads. (**C**) Viral RNA load following specified drug treatment of MHV-infected RAW264.7 cells as quantified by RT-qPCR. Synergy plot of combination dose–response along with the average Bliss synergy score for (**D**) 2-D live virus titer inhibition, and (**E**) 2D viral RNA load inhibition. Red regions indicate positive synergy scores, whereas green regions depict negative scores. The zones demarcated by the white lines reflect heightened pharmacological synergy of the drug combination.

Minimal drug toxicity was observed in RAW264.7 cells when treated by the combination of Nirmatrelvir and Ivermectin, i.e., the vast majority of the tested combinations maintained cell viability of over 80% (Figure 2A). Nirmatrelvir monotherapy at 5 μM achieved a reduction of 5 to 6 log_10_ in live virus titer, but no virus load reduction was observed for Ivermectin monotherapy. In comparison, several Nirmatrelvir–Ivermectin combinations considerably diminished live virus loads of at least 5 to 6 log_10_, such as the combination of 2.5 μM of Nirmatrelvir and 0.15 μM of Ivermectin. In addition, it is notable that live virus loads of MHV diminished in a dose-dependent manner (by up to 3 log_10_) at 1.25 μM of Nirmatrelvir combined with increasing concentrations of Ivermectin (Figure 2B). Representative images of virus plaque assays are illustrated in Supplementary Figure S1.

Similarly, but to a lower extent, the viral RNA levels also decreased in a dose-dependent manner at 1.25 μM Nirmatrelvir combined with increasing concentrations of Ivermectin (Figure 2C). When assessed by SynergyFinder 3.0, the Nirmatrelvir and Ivermectin checkerboard assay yielded an average Bliss synergy score of 9.95 for live virus titer (Figure 2D) and of 7.41 for viral RNA load (Figure 2E). These values suggest strong synergism between the two drugs in combination. Based on the Bliss inhibition synergy plot of the drug combination on both live virus titer and viral RNA load, the most synergistic concentrations ranged from 1.25 μM to 2.5 μM of Nirmatrelvir, and from 0.6 μM to 2.4 μM of Ivermectin.

To ensure reproducibility of the results, a biological replicate experiment of the checkerboard assay revealed similar synergistic effects, e.g., 2.5 μM of Nirmatrelvir combined with 0.6 μM of Ivermectin could attain a 7-log_10_ reduction in live virus titer and a 3-log_10_ reduction in viral RNA load.

### 2.3. Combination Therapy of Nirmatrelvir and Azithromycin Exhibits Additive Effects on MHV Inhibition

**Figure 3:**
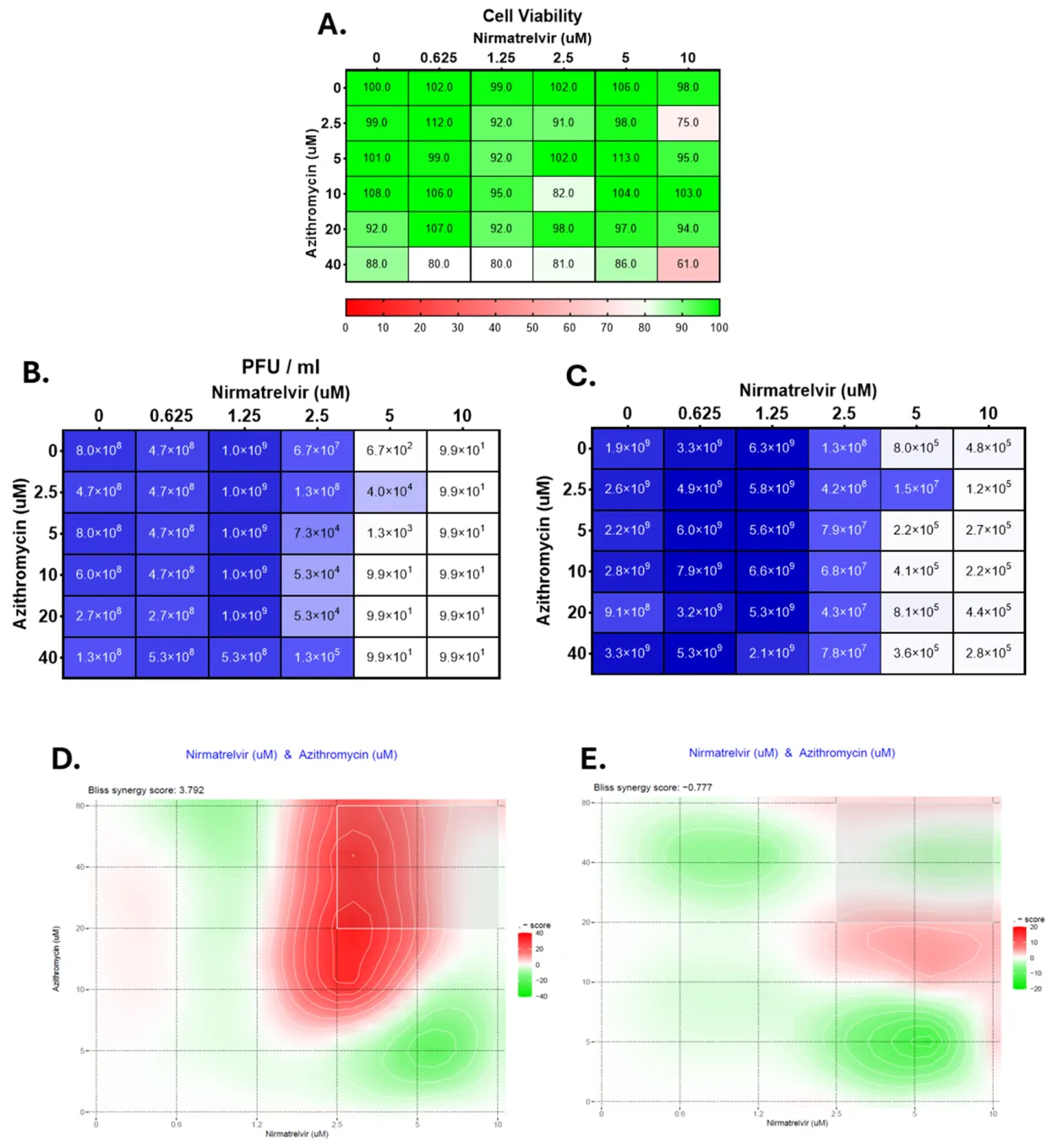
Checkerboard assays of the Nirmatrelvir and Azithromycin combination treatment of RAW264.7 cells. (**A**) Cytotoxicity profile of the drug combination on RAW264.7 cells as measured by the MTS assay. The reference control was healthy cells (without infection and without drug treatment) with an assigned cell viability of 100%. Hence, cell viability percentages above 100% reflect viable healthy cells. Cell viability of 80% or greater is generally accepted for a drug treatment to be considered as non-cytotoxic. (**B**) Live viral titer of MHV following specified drug treatment of MHV-infected RAW264.7 cells as quantified by virus plaque assay. The value of 9.9 × 10^1^ (= 99) indicates a live virus titer of less than 100 PFU/mL. The darker shades of blue denote higher viral loads, whereas the white areas reflect very low viral loads. (**C**) Viral RNA load following specified drug treatment of MHV-infected RAW264.7 cells as quantified by RT-qPCR. Synergy plot of combination dose–response along with the average Bliss synergy score for (**D**) 2-D live virus titer inhibition, and (**E**) 2D viral RNA load inhibition. Red regions indicate positive synergy scores, whereas green regions depict negative scores. The zone demarcated by the white lines reflects statistically meaningful pharmacological enhancement by the drug combination.

In general, there was minimal drug toxicity of the Nirmatrelvir and Azithromycin combinations on RAW264.7 cells, with the majority of the combined drugs maintaining a cell viability of over 80% (Figure 3A). However, the combinations with 40 μM Azithromycin were excluded from analysis since the cell viability mostly hovered around 80%, with its combination with 10 μM Nirmatrelvir being 61% (suggesting cytotoxicity). Live virus titers were not reduced by Azithromycin monotherapy. In comparison with the untreated control, Nirmatrelvir monotherapy attained a 6-log_10_ reduction in live virus titer at 5 μM, but only a 1-log_10_ reduction at 2.5 μM. From the other checkerboard assay results, the live virus titer decreased by an additional 3 log_10_ when 2.5 μM Nirmatrelvir was combined with Azithromycin at concentrations of 5, 10 and 20 μM (Figure 3B). A similar trend was also observed for the viral RNA load in comparison with untreated control, whereby a 3-log_10_ reduction was achieved by Nirmatrelvir monotherapy at 5 μM, but no reduction by Azithromycin monotherapy. Interestingly, the viral RNA load was relatively unchanged when 2.5 μM Nirmatrelvir was combined with Azithromycin at concentrations of 5, 10, and 20 μM (Figure 3C). Overall, the Nirmatrelvir–Azithromycin checkerboard assay yielded average Bliss synergy scores of 3.79 for live virus titer (Figure 3D) and −0.78 for viral RNA load (Figure 3E), which suggests an additive effect rather than synergistic effect.

A biological replicate of combination treatment by 5 μM of Nirmatrelvir with 10 or 20 μM of Azithromycin resulted in an additional reduction in live virus titer of 2 to 3 log_10_ compared to 5 μM Nirmatrelvir alone, thereby verifying the additive effect of the drug combination.

### 2.4. Combination Therapy of Nirmatrelvir and Doxycycline Exhibits Some Additive Effect

**Figure 4:**
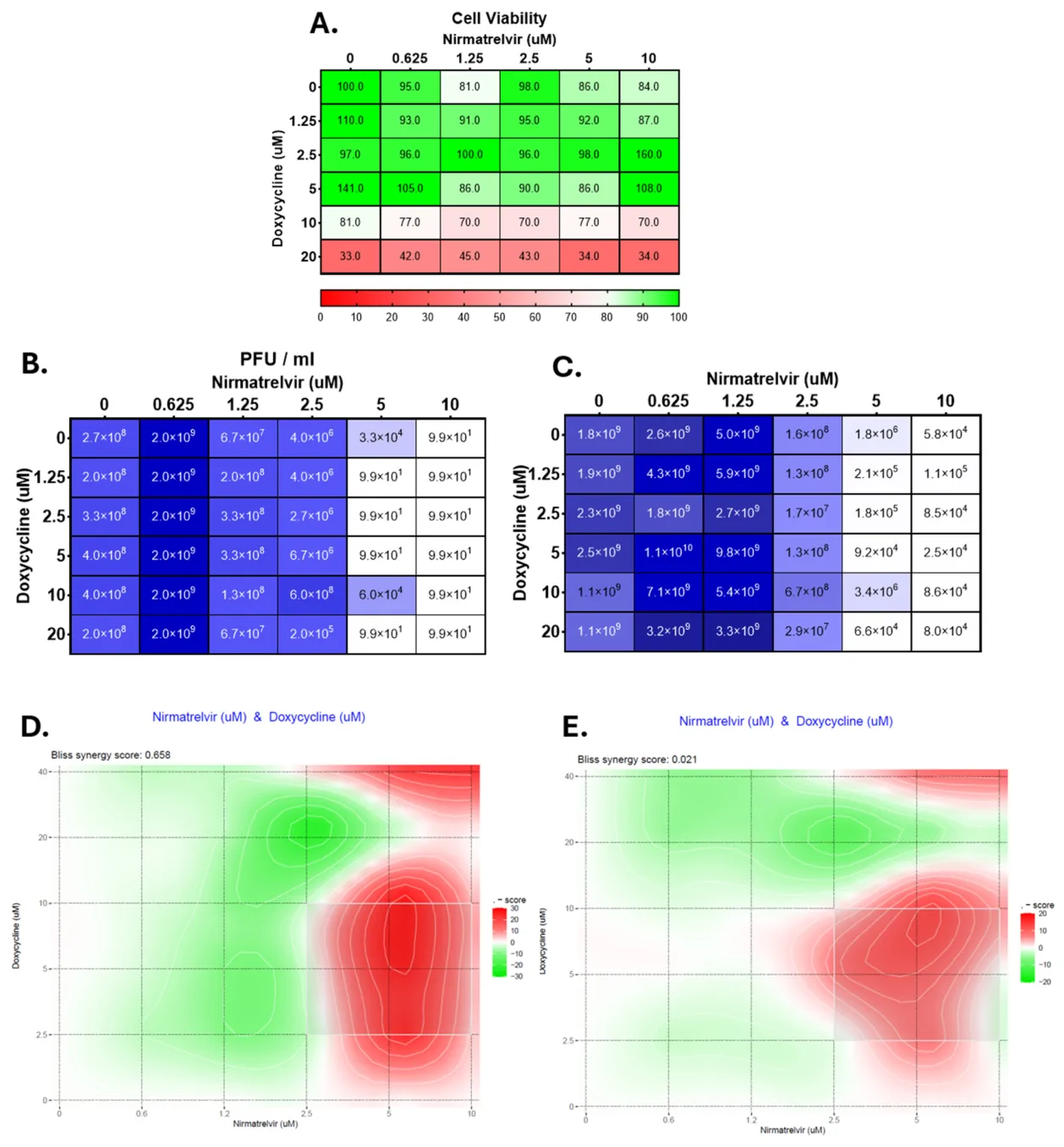
Checkerboard assays of the Nirmatrelvir and Doxycycline combination treatment of RAW264.7 cells. (**A**) Cytotoxicity profile of the drug combination on RAW264.7 cells as measured by the MTS assay. The reference control was healthy cells (without infection and without drug treatment) with an assigned cell viability of 100%. Hence, cell viability percentages above 100% reflect viable healthy cells. Cell viability of 80% or greater is generally accepted for a drug treatment to be considered as non-cytotoxic. (**B**) Live viral titer of MHV following specified drug treatment of MHV-infected RAW264.7 cells as quantified by virus plaque assay. The value of 9.9 × 10^1^ (=99) indicates a live virus titer of less than 100 PFU/mL. The darker shades of blue denote higher viral loads, whereas the white areas reflect very low viral loads. (**C**) Viral RNA load following specified drug treatment of MHV-infected RAW264.7 cells as quantified by RT-qPCR. Synergy plot of combination dose–response along with the average Bliss synergy score for (**D**) 2-D live virus titer inhibition, and (**E**) 2D viral RNA load inhibition. Red regions indicate positive synergy scores, whereas green regions depict negative scores. The zones demarcated by the white lines reflect statistically meaningful pharmacological enhancement by the drug combination.

For the drug combinations of Nirmatrelvir with Doxycycline, drug toxicity was observed in RAW264.7 cells only in combinations comprising 20 μM and 10 μM of Doxycycline which displayed cell viability values near or below 80% (Figure 4A). Therefore, these two higher Doxycycline concentrations were excluded from the analysis of the checkerboard assay data. In comparison with the untreated control, the live virus titer was diminished by 2 log_10_ and 4 log_10_ by Nirmatrelvir monotherapy at 2.5 and 5 μM respectively, whereas there was no live virus titer reduction by Doxycycline monotherapy. From the checkerboard results of the drug combinations, we observed that the live virus titer decreased by an additional 2 log_10_ when 5 μM Nirmatrelvir was combined with Doxycycline at concentrations of 1.25, 2.5, and 5 μM (Figure 4B). Compared with the untreated control, the viral RNA load was reduced by 3 log_10_ with Nirmatrelvir monotherapy at 5 μM, but no reduction was noted for Doxycycline monotherapy—revealing a similar trend as the live virus load. For the combination of 5 μM Nirmatrelvir with Doxycycline at 1.25, 2.5 and 5 μM, there was an additional reduction in viral RNA load of 1 log_10_ (Figure 4C). Overall, the Nirmatrelvir and Doxycycline checkerboard assay generated average Bliss synergy scores of 0.66 for live virus titer (Figure 4D) and 0.02 for viral RNA load (Figure 4E), which is suggestive of only mildly additive effect.

A biological replicate of the checkerboard experiment also revealed that combinations of Nirmatrelvir with 20 μM and 10 μM of Doxycycline were cytotoxic with cell viability ranging from 25% to 70%.

### 2.5. Combination Therapy with Nirmatrelvir and Ivermectin Affects the Coronavirus Replication Cycle at the Same Time-Point as Nirmatrelvir Monotherapy

**Figure 5:**
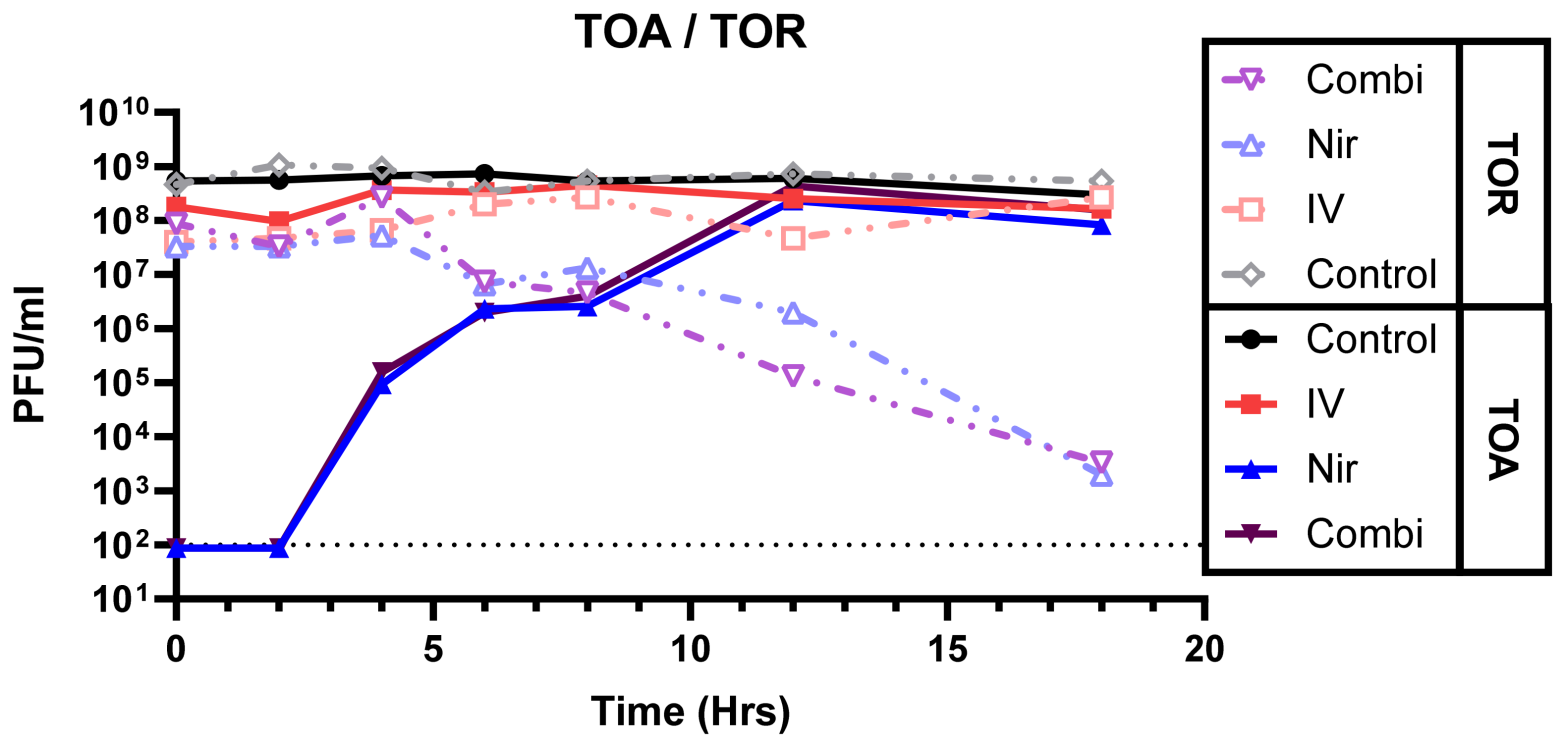
Time-of-addition (TOA) and time-of-removal (TOR) assays of drug treatments of RAW264.7 macrophages infected with MHV. Details of the TOA and TOR assays are outlined in the Materials and Methods section. Live viral titers of MHV following specified drug treatments of MHV-infected RAW264.7 cells, quantified using virus plaque assay (*y*-axis) at time-points of 0 h, 2 h, 4 h, 6 h, 8 h, 12 h, and 18 h post-infection (*x*-axis). TOA, continuous lines; TOR, broken lines; Combi, combination of Nirmatrelvir and Ivermectin; Nir, Nirmatrelvir; IV, Ivermectin; Control, no treatment. The lower dotted line indicates live viral titers of 100 PFU/mL

The time-of-addition (TOA) assay estimates the minimum time at which the drug must be added for its antiviral effect to be observed, while the time-of-removal (TOR) assay estimates the duration of exposure needed for the drug to exert its effect. The intersection of the TOA and TOR graphs provides an estimate of the critical time-period when a given drug exerts its effect on the viral replication cycle [34,35]. Based on the results of the TOA and TOR assays (Figure 5), the combination treatment of Nirmatrelvir and Ivermectin exerted its effect on viral replication at approximately 8 h post-infection. Similarly, Nirmatrelvir monotherapy exerted its effect at the similar time-period of 8 h. Nirmatrelvir inhibits post-translational processing of coronaviral pp1a and pp1ab proteins to mediate viral inhibition, congruent with the estimated time-period indicated by the TOA and TOR results [36]. The convergence in the time of action between the combination and Nirmatrelvir monotherapy suggests that combination acts in a similar fashion, albeit at a lower concentration of Nirmatrelvir. Hence, the results indicate that the addition of Ivermectin enhances the action of Nirmatrelvir, thus reducing the amount of Nirmatrelvir necessary to achieve sufficient viral inhibition.

### 2.6. Combination Therapy with Nirmatrelvir and Ivermectin Significantly Suppresses the Levels of Key Cytokines and Chemokines Associated with Cytokine Storm

Given that the Nirmatrelvir–Ivermectin combination could effectively inhibit live virus and viral RNA, we next analyzed the drug combination’s effects on the protein expression levels of a panel of 26 cytokines in infected macrophages.

**Figure 6:**
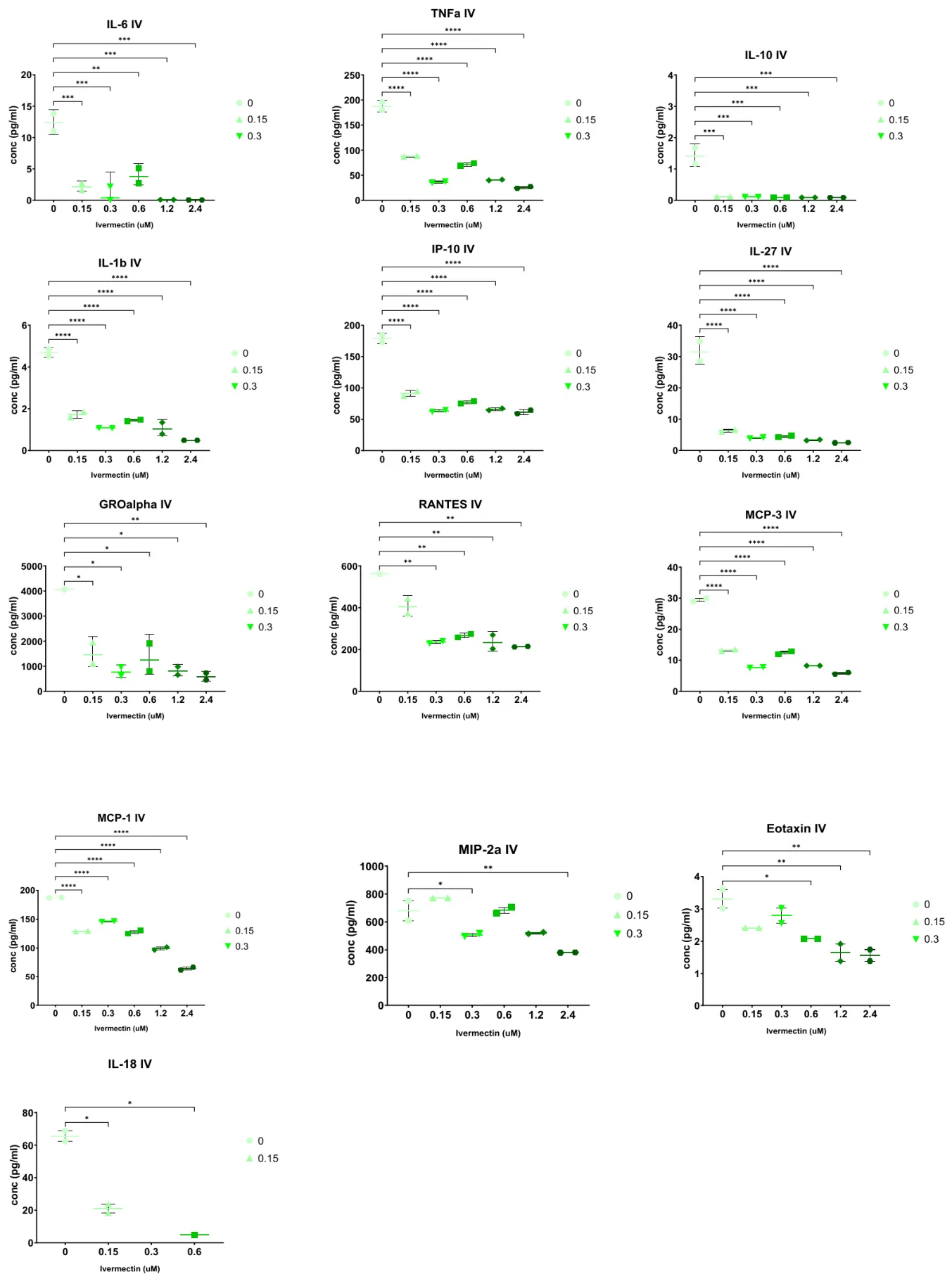
Cytokine and chemokine protein expression profiles of Nirmatrelvir and Ivermectin combination therapy against MHV infection of RAW264.7 macrophages. The *y*-axis shows the cytokine or chemokine concentration (pg/mL). The *x*-axis shows various Ivermectin (IV) concentrations (from 0 to 2.4 μM) combined with 2.5 μM Nirmatrelvir. Each green horizontal line indicates the mean protein concentration along with the standard error of the mean represented by an error bar. Statistical significance was determined by one-way ANOVA with Bonferroni’s multiple comparison tests against the untreated infected control. * *p* < 0.05; ** *p* < 0.01; *** *p* < 0.001; **** *p* < 0.0001.

Combination treatment with Nirmatrelvir and Ivermectin was found to significantly decrease 13 cytokines and chemokines (Figure 6). The concentrations of key pro-inflammatory cytokines such as IL-6 decreased to undetectable range, while significant reductions were also observed for TNF-α (7.5-fold), IL-1β (9.3-fold), and MCP-1 (3-fold). The levels of IL-10, IL-27, GRO-alpha, RANTES, MCP-3, MIP-2, Eotaxin, and IL-18 were also downregulated compared to the untreated infected control. The levels of the other analytes that showed no significant differences are shown in Appendix A.

Since IL-6 is one of the most important cytokines in COVID-19 pathogenesis, we also quantified mRNA expression of IL-6 in MHV-infected RAW264.7 cells for the checkerboard assay of Nirmatrelvir and Ivermectin treatment. Compared to the untreated infected control, the relative IL-6 mRNA expression was relatively unchanged for monotherapy with either Nirmatrelvir or Ivermectin. In contrast, combination treatment with Nirmatrelvir and Ivermectin generally diminished IL-6 mRNA expression by up to 5-fold compared to the control. Notably, the combination of 1.2 μM of Ivermectin with various concentrations of Nirmatrelvir consistently depressed IL-6 mRNA expression by three to four fold compared to control (Figure A4). This further supports the downregulation of IL-6 at both translational and transcriptional levels by the Nirmatrelvir–Ivermectin combination.

It is noteworthy that the Nirmatrelvir–Ivermectin combination could attain at least 5-log_10_ reduction in live virus titer (Figure 2B), which is complemented by amelioration of cytokine production to exert strong anti-inflammatory effects. Especially relevant are IL-6, TNF-α and IL-1β which are significantly elevated in patients with severe COVID-19 and contribute to the pathogenesis of the cytokine storm [37,38,39].

### 2.7. Combination Treatments of Nirmatrelvir Paired with Doxycycline or Azithromycin Display Some Anti-Inflammatory Effects

**Figure 7:**
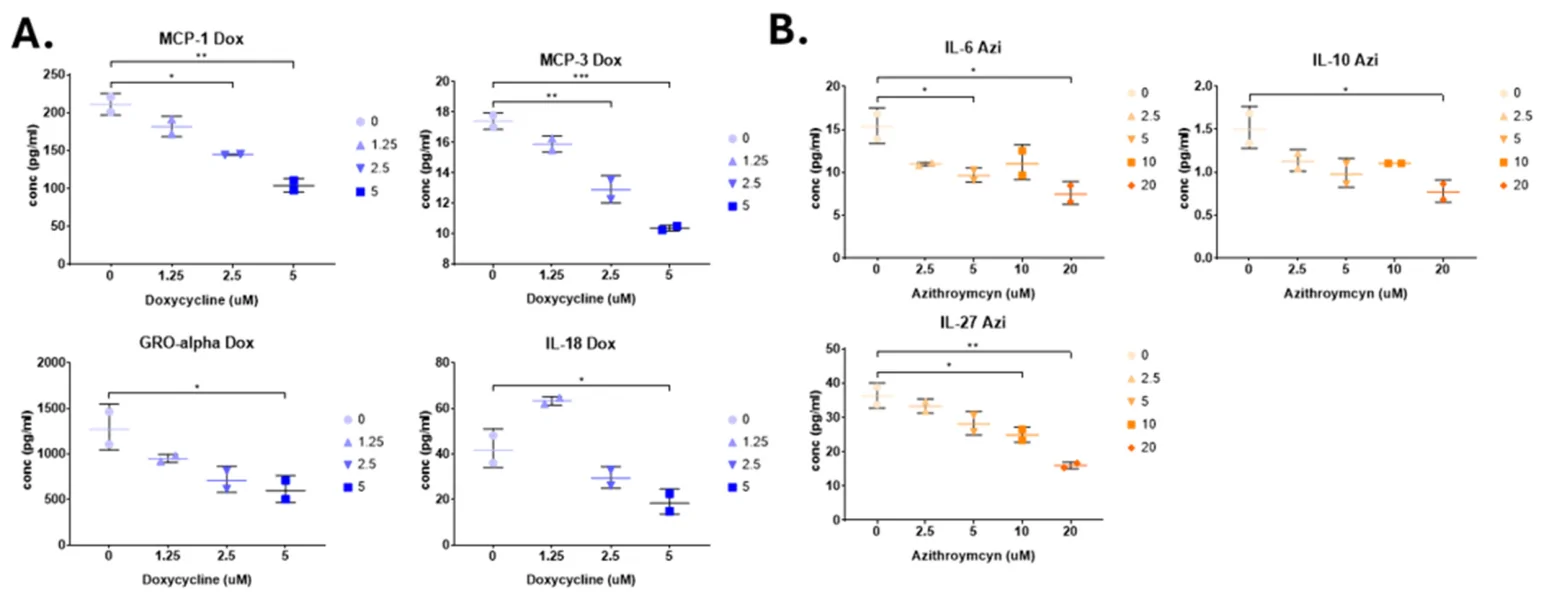
Cytokine and chemokine protein expression profiles of Nirmatrelvir–Doxycycline and Nirmatrelvir–Azithromycin combination treatments against MHV infection of RAW264.7 macrophages. The *y*-axis shows the cytokine or chemokine concentration (pg/mL). (**A**) The *x*-axis shows various Doxycycline (Dox) concentrations (from 0 to 5 μM) combined with 5 μM Nirmatrelvir. (**B**) The *x*-axis shows various Azithromycin (Azi) concentrations (from 0 to 20 μM) combined with 2.5 μM Nirmatrelvir. Each colored horizontal line indicates the mean protein concentration along with the standard error of the mean represented by an error bar. Statistical significance was determined by one-way ANOVA with Bonferroni’s multiple comparison tests against the untreated infected control. * *p* < 0.05; ** *p* < 0.01; *** *p* < 0.001.

**Figure A3:**
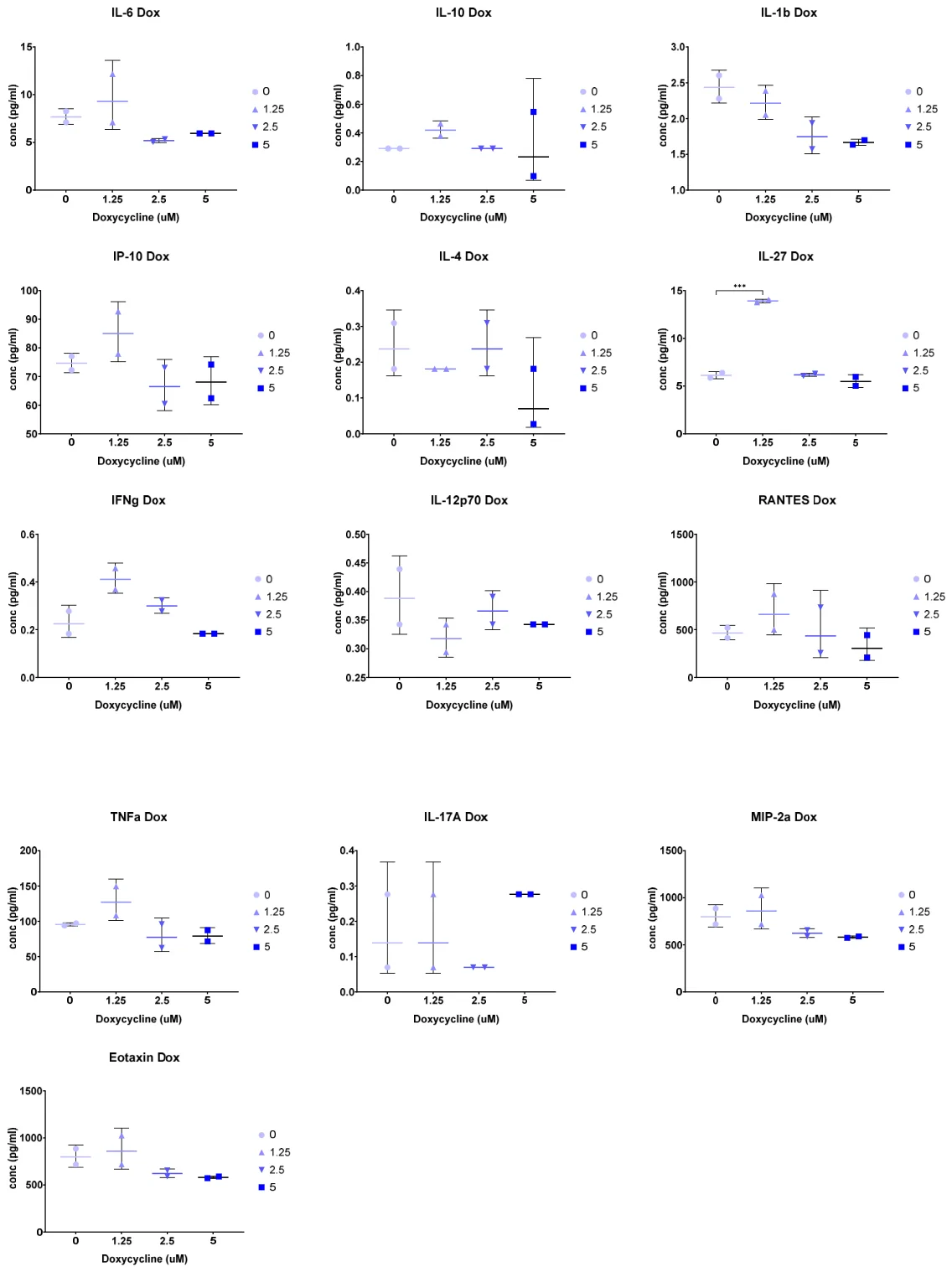
Cytokine and chemokine expression protein profiles of Nirmatrelvir and Doxycycline combination treatment against MHV infection of RAW264.7 macrophages. The *y*-axis shows the cytokine or chemokine concentration (pg/mL). The *x*-axis shows various Doxycycline (Dox) concentrations (from 0 to 5 μM) combined with 5 μM Nirmatrelvir. Each blue horizontal line indicates the mean protein concentration along with the standard error of the mean represented by an error bar. There was no statistical significance as determined by one-way ANOVA with Bonferroni’s multiple comparison tests against the untreated infected control (except for IL-27 with *** *p* < 0.001). IL-2, IL-5, IL-9, IL-13, IL-22, IL-23, and GM-CSF were not detected. MIP-1-alpha and MIP-1-beta concentrations were beyond the upper limit of quantification.

**Figure A4:**
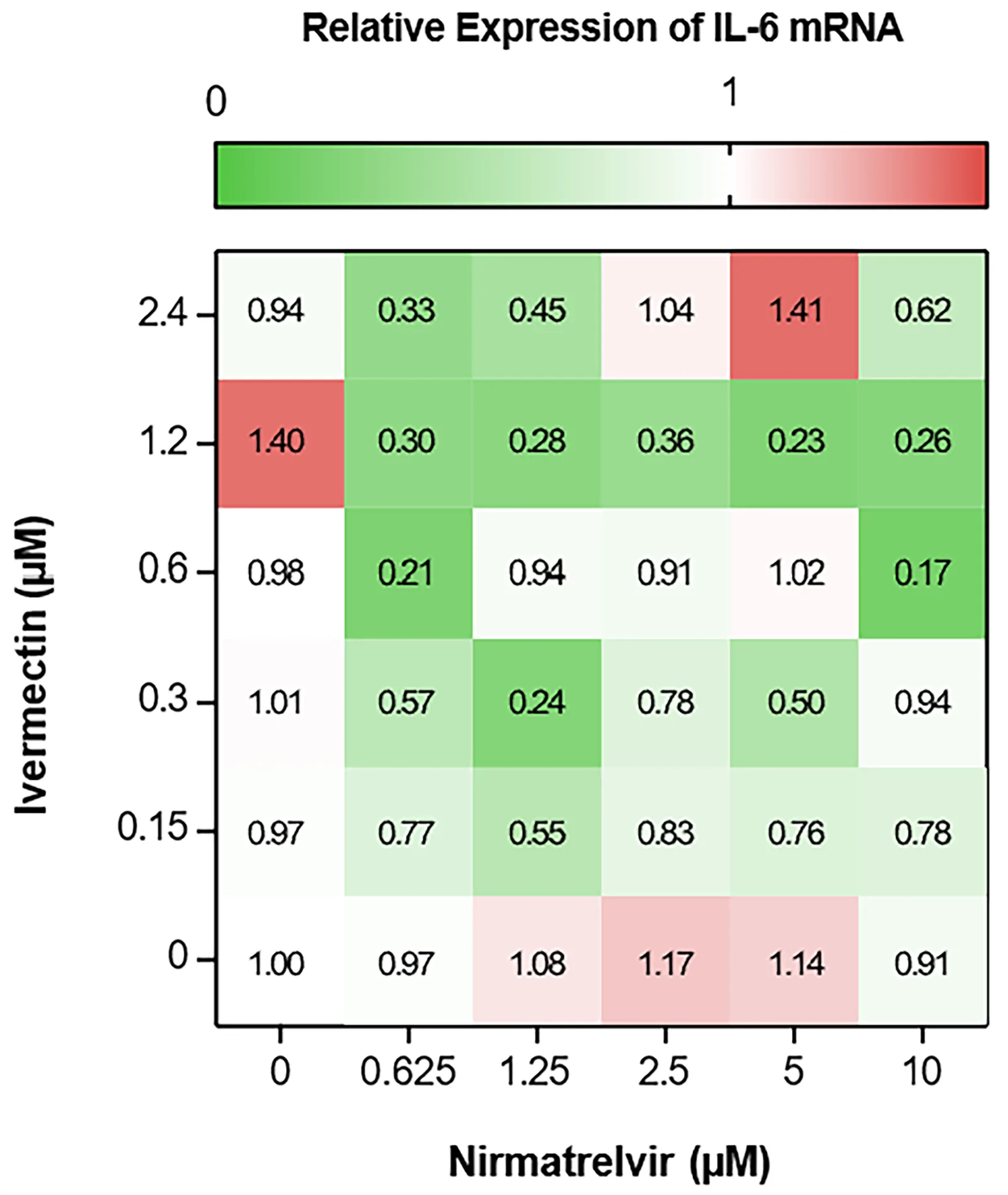
Heatmap of the relative IL-6 mRNA expression of MHV-infected RAW264.7 cells for the checkerboard assay of Nirmatrelvir and Ivermectin. IL-6 mRNA expression was analyzed by RT-qPCR, and the fold change in gene expression was calculated relative to the untreated infected control (assigned a value of 1). Relative expression of <1 indicates reduced IL-6 mRNA expression, whereas a value of >1 indicates increased expression. The deeper shade of green depicts greater reduction, while the deeper shade of red denotes greater increase in relative IL-6 mRNA expression.

Combination treatment with Nirmatrelvir and Doxycycline was found to decrease IL-18, MCP-1, MCP-3, and GRO-alpha (all statistically significant at *p* < 0.05) (Figure 7A). However, Nirmatrelvir–Doxycycline did not significantly reduce IL-6 and TNF-α (Figure A3) which are important pro-inflammatory cytokines in the cytokine storm. Combination treatment with Nirmatrelvir and Azithromycin decreased IL-6, IL-10 and IL-27 (all statistically significant at *p* < 0.05) (Figure 7B). On the other hand, the other cytokine and chemokine analytes showed no significant difference compared to the untreated infected control, as shown in Appendix A. The findings suggest that these two drug combinations possess some anti-inflammatory effects.

## 3. Discussion

The objective of this study was to identify potentially efficacious dual-drug combination therapy involving Nirmatrelvir and three selected repurposed drugs. These therapies are hypothesized to exert antiviral effects by targeting both the virus and the host, while also possessing immunomodulating properties. We employed the model of MHV infection of the RAW264.7 cell line, which is an adherent mouse macrophage cell model widely used for drug development, inflammatory, and immune response studies [20]. The study initially investigated the efficacy of each drug as monotherapy before proceeding to checkerboard assays to assess combination treatment efficacy. To increase confidence and to ensure the reproducibility of data, each checkerboard assay was repeated as an additional biological replicate to obtain more robust results. Additionally, immunomodulating effects such as the downregulation of pro-inflammatory cytokines and chemokines were also investigated for the combination therapies.

An ideal synergistic drug combination should achieve a reduction in live viral load to below 100 PFU/mL, while showcasing an average Bliss synergy score exceeding 10. Moreover, the synergistic drug combination should also demonstrate minimal toxicity towards RAW264.7 cells. These important criteria were generally fulfilled by the Nirmatrelvir-Ivermectin combination with a predicted Bliss synergy score of 9.95. An additional reduction of at least 2 log_10_ in live viral load was contributed by the synergistic effects of the drug combination. Repeat experiments indeed confirmed that these strongly synergistic effects were reproducible. Importantly, minimal toxicity was also observed with this combination, suggesting the absence of toxic drug–drug interactions within the cellular context of RAW264.7 cells.

Interestingly, we noticed a relatively higher reduction in live virus titer than the magnitude of reduction in viral RNA load. This difference in reduction may be attributed to the fact that the virus produces more RNA copies than necessary, and that not all the viral RNA will be translated into viral proteins to generate viable whole virus. Infected host cells release mature replication-competent virions along with abundant defective viral particles and loose RNA transcripts. Indeed, total coronavirus RNA levels in COVID-19 patient samples are typically significantly greater than their titers of live infectious virus. This is attributed to the RT-qPCR assay detecting RNAs from both infectious intact virions as well as non-infectious incomplete virions and viral fragments [40,41,42].

Moreover, when combined with Ivermectin, a two-fold lower concentration of Nirmatrelvir could achieve the same inhibitory effect on live virus as 5 μM Nirmatrelvir monotherapy. This suggests the potential for using lower Nirmatrelvir dosages in combination with Ivermectin. In the Nirmatrelvir–Ivermectin combination, complete live virus inhibition was achieved with Nirmatrelvir at a concentration lower than its IC50, and with Ivermectin at a concentration that is lower than that in its combination with Remdesivir [20,21].

The parallel synergistic effects of the Remdesivir and Ivermectin combination against both MHV and SARS-CoV-2 strengthens the notion that MHV serves as a valuable model for testing drugs against human *Betacoronaviruses*, particularly SARS-CoV-2 [20,21]. Furthermore, the minimal toxicity of this Nirmatrelvir–Ivermectin combination in RAW264.7 cells suggests that it is unlikely to result in harmful drug–drug interactions, and has promising therapeutic potential.

Ivermectin demonstrated strong synergism in combination with different antivirals, i.e., Remdesivir or Nirmatrelvir, despite their differences in mechanism of antiviral action. This suggests that Ivermectin can exert its synergistic antiviral effects via mechanisms that complement the diverse modes of action of these antivirals, potentially rendering Ivermectin as a beneficial component in specific drug combination regimens against coronaviruses.

Time-of-addition and time-of-removal assays were conducted to pinpoint the phase of the MHV replication cycle targeted by either the monotherapies or combination treatment. By plotting live viral load against time for these assays, the time-point at which the live viral load curves intersect provides crucial information on when the therapy acts, i.e., either during the early (e.g., entry) or late (e.g., post-entry) phases of the viral life-cycle. This helps to deduce the mechanism of action of the combination therapy relative to monotherapy. It is noteworthy that the Nirmatrelvir–Ivermectin combination treatment affected the coronavirus replication cycle at the same time-point of 8 h as Nirmatrelvir monotherapy, thus implying that they share similar post-entry phase target(s).

In this study, Nirmatrelvir and Ivermectin exhibited highly synergistic effects in strongly inhibiting live viral load and downregulating the expression of pro-inflammatory cytokines such as IL-6. The identification of a highly synergistic combination involving an oral antiviral against SARS-CoV-2 (Nirmatrelvir) and a repurposed drug (Ivermectin) offers a promising approach for improving the current treatment modalities. Advantages of this combination would include reducing the Nirmatrelvir dose, and preventing severe disease progression by attenuating any cytokine storm.

Notably, the synergistic Nirmatrelvir–Ivermectin combination therapy with both antiviral and anti-inflammatory activities represent novel findings, particularly involving the use of oral drugs. This study is supported by experimental evidence indicating antiviral efficacy, and further strengthens the notion that combination therapy is highly promising as a therapeutic strategy. In our study, the utilization of repurposed drugs for combination therapy has alluded to the application of approved drugs for new indications.

By quantifying cytokine protein expression using Bio-Plex cytokine assays, we observed significant downregulation of 13 cytokines and chemokines in response to combination treatment with Nirmatrelvir and Ivermectin. For example, there was a consistent and marked reduction in IL-6 protein levels observed across various drug doses. This suggests strong immunomodulating effects of the Nirmatrelvir–Ivermectin combination in downregulating pro-inflammatory cytokines such as IL-6. Downregulating IL-6 expression is crucial in preventing severe disease progression, since it is a critical inflammatory cytokine implicated in the cytokine storm [43,44,45]. The cytokine storm is a hyperinflammatory state characterized by excessive production of cytokines by dysregulated immune responses. It is one of the major causes for serious complications such as coagulopathy, multi-organ failure, and even death [46]. Studies show the critical role of the cytokine storm in COVID-19 patients, with elevated levels of key pro-inflammatory cytokines (such as IL-1β, IL-2, IL-6, TNF-α, IFN-γ, IP-10, GM-CSF, MCP-1), which correlate with disease severity [47].

In contrast, for the Nirmatrelvir combinations with Azithromycin or Doxycycline, we observed only additive or mildly additive effects, albeit with some immunomodulatory effects. Thus, Nirmatrelvir–Azithromycin downregulated IL-6 and IL-10, whereas Nirmatrelvir–Doxycycline did not downregulate key cytokines such as IL-6 and TNF-α. This reiterates that certain drug combination treatments cannot potently decrease viral infection, and are unable to significantly modulate cytokine expression. These varying effects and outcomes can be attributed to their different mechanisms of action and interactions.

In the current and future context, it is important to identify and deploy efficacious antiviral drug combinations involving the use of oral antivirals for the treatment of coronavirus infections. SARS-CoV-2 is proving to be resilient, since its infection has become endemic globally, and its complete eradication is quite unlikely in the future [48]. The unrelenting emergence of new SARS-CoV-2 variants, driven by the inherently high mutation rate of coronaviruses, results in variants exhibiting a spectrum of transmissibility and virulence [1]. While vaccination efforts have been instrumental in reducing the severity of illness and transmission rates, they may not offer full protection against evolving variants. Consequently, the need for booster vaccines persists [49]. Moreover, certain high-risk groups (e.g., the elderly and immunocompromised individuals) may not respond as effectively to COVID-19 vaccines, thus potentially rendering them more vulnerable to severe illness from the virus. Therefore, the availability of effective antiviral treatments against SARS-CoV-2 becomes even more crucial for disease management, especially in mitigating the progression towards severe disease [50].

Moreover, the past two decades has witnessed the emergence of several deadly and highly pathogenic human *Betacoronaviruses*, indicating the ever-increasing threat of zoonotic spillover events culminating in pandemics. This trend is exacerbated by urbanization and human expansion into previously uninhabited areas such as forests or wetlands, which can increase the likelihood of contact between humans and wildlife that harbor infectious agents. This heightened interaction raises the risk of zoonotic pathogen transmission from animals to humans [51,52,53]. Hence, proactive pre-pandemic preparation with antiviral options capable of targeting similar human coronaviruses is imperative to mitigate the impact of future pandemics. In an event of a pandemic, oral antivirals administered outside of hospital settings play critical roles in virus control and disease management, particularly in preventing disease progression to severe outcomes. Oral antivirals such as Paxlovid and Molnupiravir hold promise as potential options for future outbreaks of similar human coronaviruses. However, treatments with these drugs in the context of COVID-19 can be further improved.

This study has certain limitations. One limitation of the checkerboard assays was the use of a restricted set of concentrations for drug testing to establish dose–response curves for both cytotoxicity and viral inhibition. This limited dataset may potentially hinder the detection of maximal synergistic effects conferred by other drug concentrations. To specifically address this, future experimental design can be refined by incorporating a broader and more diverse range of drug concentrations in checkerboard assays. Such an adjustment would facilitate a more precise determination of parameters such as virus inhibition and immunomodulation, and provide a more thorough and comprehensive understanding of combined drug potency. Further studies should also be expanded to explore potential synergistic effects of combining Nirmatrelvir and other antivirals (e.g., Molnupiravir) with other repurposed drugs.

The mechanism of action of combination therapy may also be elucidated using the approaches of proteomics, transcriptomics, and interactomics. Proteomics enables the identification and quantification of critical viral and host proteins that may be modulated by the therapy, which potentially mediates inhibition of viral replication and/or modulation of host responses. Transcriptomics involves gene expression profiling, and offers insights into alterations in gene expression patterns induced by the therapy. This approach serves as a complementary validation to proteomics, offering a comprehensive view of the molecular mechanisms underlying the therapeutic effects [21,54]. Interactomics focuses on identifying drug–target protein interactions, and helps to pinpoint specific proteins targeted by the therapy [55]. These interactions may provide valuable insights into the molecular pathways impacted by the therapy and its molecular mode(s) of action.

The possibility of MHV developing resistance to the combination therapy can be investigated through in vitro serial passaging of MHV with Nirmatrelvir monotherapy and with Nirmatrelvir–Ivermectin combination. Following serial passaging of MHV, the viral genome can be sequenced to identify mutations in potentially drug-resistant mutant viruses. Recombinant MHV harboring specific mutations can then be generated, and treated with Nirmatrelvir monotherapy and the Nirmatrelvir–Ivermectin combination to characterize therapy-resistant mutations [56]. By comparing the number of resistant mutations between monotherapy and combination therapy, it can be determined whether the drug combination can effectively abrogate viral resistance.

To validate the results of our in vitro study on MHV, future studies are clearly warranted to evaluate whether this drug combination possesses synergistic antiviral effects against SARS-CoV-2 and other coronaviruses via in vitro experiments before progressing to animal studies, and eventually to clinical trials [57,58].

## 4. Materials and Methods

### 4.1. Virus and Cell Cultures

All viral infection and drug treatment experiments were conducted using the RAW264.7 murine macrophage cell line (GenBank accession number U29396) infected with MHV strain A59 (MHV-A59; GenBank accession number AY910861). The RAW264.7 cell line and MHV strain A59 were obtained from the American Type Culture Collection (Manassas, VA, USA). RAW264.7 cells were maintained at 37 °C in RPMI-1640 medium supplemented with 10% fetal bovine serum (FBS).

All virus plaque assay experiments were conducted using H2.35 murine liver cells, which were maintained at 35 °C in Dulbecco’s modified Eagle medium (DMEM) with 10% FBS. The H2.35 mouse hepatocyte cell line (catalog# CRL-1995; American Type Culture Collection) is widely utilized in biomedical research including characterization of pathogens and antimicrobial testing.

### 4.2. Drugs

Azithromycin (catalog# HY-17506; MedChemExpress, Monmouth Junction, NJ, USA), Ivermectin (CAS 70288-86-7; Merck, Burlington, MA, USA), and Nirmatrelvir (catalog# HY-138687; MedChemExpress) were prepared as 10 mM stock solutions in dimethyl sulfoxide (DMSO). Doxycycline (CAS 24390-14-5; Merck, Burlington, MA, USA) was prepared as 10 mM stock solution in sterile water. Drugs were diluted in 0.5% DMSO with RPMI-1640 medium supplemented with 2% FBS for all treatment experiments.

### 4.3. Cell Viability Assay

RAW264.7 cells were incubated at 35 °C in the presence of different drug concentrations for 48 h. The CellTiter 96 AQueous One Solution Cell Proliferation (MTS) reagent (Promega, Madison, WI, USA) was added to each well, and incubated at 35 °C for 15–30 min according to the manufacturer’s protocol. Absorbance readings were taken using a microplate reader (Tecan, Mannedorf, Switzerland), with values normalized to those of control untreated cells to determine the cell viability [20,21].

### 4.4. Evaluation of Antiviral Inhibitory Activities of Monotherapy and Combination Therapy

Monolayers of RAW264.7 cells (80 to 90% confluent) in 24-well plates were infected with MHV at multiplicity of infection (MOI) of 0.1 for 1 h. The virus inoculum was then discarded, without any washing steps. This continuous inoculum during drug treatment was to mimic authentic host infection of continuous exposure of host cells which are constantly in contact with viral particles. The cells were then treated with different concentrations of drugs for monotherapy or combination treatment for 48 h. The cell supernatant was harvested and subjected to plaque assay to determine live coronavirus titers, and real-time RT-qPCR to quantify the viral RNA loads [20].

### 4.5. Time-of-Addition and Time-of-Removal Assays

Sub-confluent monolayers of RAW264.7 cells in 24-well plates were infected with MHV at MOI of 0.1 for 1 h. Time-of-addition (TOA): The inoculum was then removed, and fresh medium was added. At 0, 2, 4, 6, 8, 12, and 18 h post-infection, the medium was removed, and the various drug concentrations were added. The culture supernatant was harvested at 24 h post-infection, and subjected to a virus plaque assay to determine the live coronavirus titer. Time-of-removal (TOR): The inoculum was then removed, and various drug concentrations were added. At 0, 2, 4, 6, 8, 12, and 18 h post-infection, the medium with drugs was removed, and fresh medium was added. The culture supernatant was harvested at 24 h post-infection, and subjected to a virus plaque assay to determine the live coronavirus titer [21].

### 4.6. Live Coronavirus Quantification by Virus Plaque Assay

Sub-confluent H2.35 cells were infected with serially-diluted culture supernatant samples for 1 h. Avicel BioPolymer (1.2%; FMC, Philadelphia, PA, USA) in phosphate-buffered saline (PBS) was added to each well, and incubated for 72 h. The cells were fixed with 4% paraformaldehyde in PBS for 2 h, and then stained with 1% crystal violet for 15 min before counting the plaques.

### 4.7. RNA Extraction, Quantification of Viral RNA Load, and IL-6 mRNA Expression by Real-Time Reverse Transcription-Quantitative Polymerase Chain Reaction (RT-qPCR)

Total viral RNA was purified from the harvested supernatant after treatment using the QIAamp Viral RNA Mini kit (Qiagen, Hilden, Germany). RNA concentration was determined using the NanoDrop 1000 spectrophotometer (Thermo Fisher Scientific, Waltham, MA, USA). Reverse transcription was carried out at 35 °C for 1 h. Quantification of viral RNA was performed using MHV-NF forward primer (5′-ACGCTTACATTATCWACTTC-3′) and MHV-NR reverse primer (5′-GATCTAAATTAGAATTGGTC-3′) for the specific detection of the MHV N gene. The qPCR was performed with the FastStart Essential DNA Green Master mix using the LightCycler 96 instrument (Roche Diagnostics, Basel, Switzerland). To plot the standard curve, a range of positive controls with known plaque-forming units (PFU) of MHV was included. The thermal cycling conditions were as follows: 95 °C for 10 min, followed by 55 cycles each of 95 °C for 10 s, 40 °C for 5 s, 72 °C for 8 s, and a final 95 °C for 10 s, 65 °C for 60 s, and 97 °C for 1 s.

The cDNA samples derived from prior reverse transcription were subjected to qPCR using IL-6 forward primer (5′-TAGTCCTTCCTACCCCAATTTCC-3′) and reverse primer (5′-TTGGTCCTTAGCCACTCCTTC-3′). RPL13A served as the reference housekeeping gene using RPL13A forward primer (5′-TACCAGAAAGTTTGCTTACCTGGG-3′) and reverse primer (5′-TGCCTGTTTCCGTAACCTCAAG-3′). The qPCR reaction components were the same as previously described, except with the following thermocycling parameters: preincubation at 95 °C for 10 min, followed by 45 cycles each consisting of denaturation at 95 °C for 10 s, annealing at 50 °C for 10 s, and elongation at 72 °C for 10 s, with a final step of 95 °C for 10 s, 65 °C for 60 s and 97 °C for 1 s for melt curve analysis. Each sample was analyzed in duplicate, and the fold change in IL-6 mRNA expression was determined using the formula of 2^−∆∆Ct^.

### 4.8. Determining Cytokine and Chemokine Protein Expression Profiles Using Luminex Multiplex Assay

The concentrations of a panel of 26 cytokines and chemokines in RAW264.7 cell culture supernatants were measured using the ProcartaPlex Mouse Cytokine & Chemokine Panel 1 26plex kit (Thermo Fisher Scientific, Waltham, MA, USA). The assay was conducted according to the manufacturer’s instructions with some modification whereby the wash buffer was prepared using PBS with 2% bovine serum albumin and 0.1% Tween20 (Promega, Madison, WI, USA). This 26plex kit quantified the following cytokines and chemokines: GM-CSF, IFN-gamma, IL-1-beta, IL-2, IL-4, IL-5, IL-6, IL-12p70, IL-13, IL-18, TNF-alpha, IL-9, IL-10, IL-17A (CTLA-8), IL-22, IL-23, IL-27, Eotaxin (CCL11), GRO-alpha (CXCL1), IP-10 (CXCL10), MCP-1 (CCL2), MCP-3 (CCL7), MIP-1-alpha (CCL3), MIP-1-beta (CCL4), MIP-2, and RANTES (CCL5). Standard curves and values were measured using the Luminex Bio-Plex 200 instrument (Bio-Rad, Hercules, CA, USA) and calculated using the Luminex xPONENT 4.2 software (with 50 beads being set as the detection limit) [20].

### 4.9. Statistical Analyses

The Bliss independence model was utilized to evaluate any synergetic effect in each checkerboard assay of drug combinations. This model adopts probabilistic independence, where the expected combination response is computed as the product of the individual drug responses [59]. The average Bliss synergy score of the entire dose–response matrix was calculated using SynergyFinder 3.0 [19,60,61]. Statistical significance and *p*-values were calculated by one-way ANOVA using Bonferroni’s multiple comparison test via GraphPad Prism 10 software (GraphPad, San Diego, CA, USA). Each error bar of cytokine or chemokine concentration represents the standard error of geometric mean (SEM) of two technical replicates.

## 5. Conclusions

In summary, this study demonstrates that the combination of Nirmatrelvir and Ivermectin exerts potent synergistic antiviral activity against MHV infection of RAW264.7 macrophages, achieving substantial reductions in viral titer and RNA load at lower drug concentrations than Nirmatrelvir monotherapy. This drug combination also markedly attenuated key pro-inflammatory cytokines and chemokines implicated in hyperinflammation associated with COVID-19. Thus, IL-6 was diminished to undetectable levels, while TNF-α, IL-1β, IL-10, and MCP-1 levels were significantly downregulated. This anti-inflammatory activity will be useful in mitigating the cytokine storm which contributes to disease severity. These findings highlight both the antiviral and immunomodulatory properties of combining Ivermectin with Nirmatrelvir, similar to the synergistic effects of combining Ivermectin with Remdesivir. This emphasizes the synergistic potential of the combination treatment strategy of pairing selected antiviral agents with specific repurposed drugs.

By repurposing Ivermectin together with Nirmatrelvir which targets the key 3CL protease enzyme that is highly conserved across all coronaviruses, this drug combination represents a potentially promising therapeutic strategy against SARS-CoV-2 and other coronaviruses. This study underscores the potential promise of combination therapy as a strategy to enhance antiviral efficacy while mitigating cytokine-mediated pathology. This work also provides a mechanistic basis and foundation for further follow-up studies. In vivo validation using animal models (e.g., human angiotensin-converting enzyme 2 transgenic mice, hamster, ferret, and non-human primate models) will be essential to further confirm therapeutic efficacy and safety, while evaluation against SARS-CoV-2 and other coronaviruses would further strengthen clinical relevance [62,63,64]. Should positive preclinical data be forthcoming, this drug combination may progress to clinical trials to evaluate its treatment safety and efficacy against COVID-19 and other coronavirus infections.

## Figures and Tables

**Table 1:** Summary of CC50, IC50 and selectivity index values of individual drugs in RAW264.7 cells and MHV infection. CC50 and IC50 were calculated using GraphPad Prism, after fitting of their respective dose–response curves. Selectivity index (SI) was calculated with the following equation: SI = CC50 divided by IC50.

	Drug	CC50 (μM)	IC50 (μM)	Selectivity Index (SI)
Antiviral	Nirmatrelvir	66.09	6.68	9.89
Repurposed Drugs	Azithromycin	156.3	21.79	7.17
	Doxycycline	18.37	10.47	1.75
	Ivermectin	2.82	0.70	4.03

**Figure A1:**
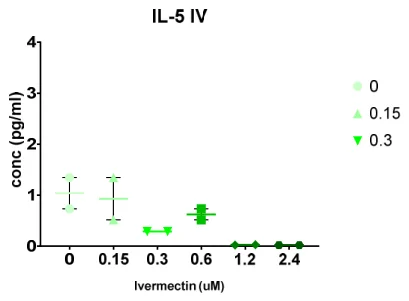
IL-5 cytokine protein expression profile of Nirmatrelvir and Ivermectin combination treatment against MHV infection of RAW264.7 macrophages. The *y*-axis shows the IL-5 concentration (pg/mL). The *x*-axis shows various Ivermectin (IV) concentrations (from 0 to 2.4 μM) combined with 2.5 μM Nirmatrelvir. Each green horizontal line indicates the mean IL-5 concentration along with the standard error of the mean represented by an error bar. There was no statistical significance as determined by one-way ANOVA with Bonferroni’s multiple comparison tests against the untreated infected control. IL-2, IL-4, IL-9, IL-13, IL-22, IL-23, IL-12p70, IL-17A, IFN-gamma and GM-CSF were not detected. MIP-1-alpha and MIP-1-beta concentrations were beyond the upper limit of quantification.

**Figure A2:**
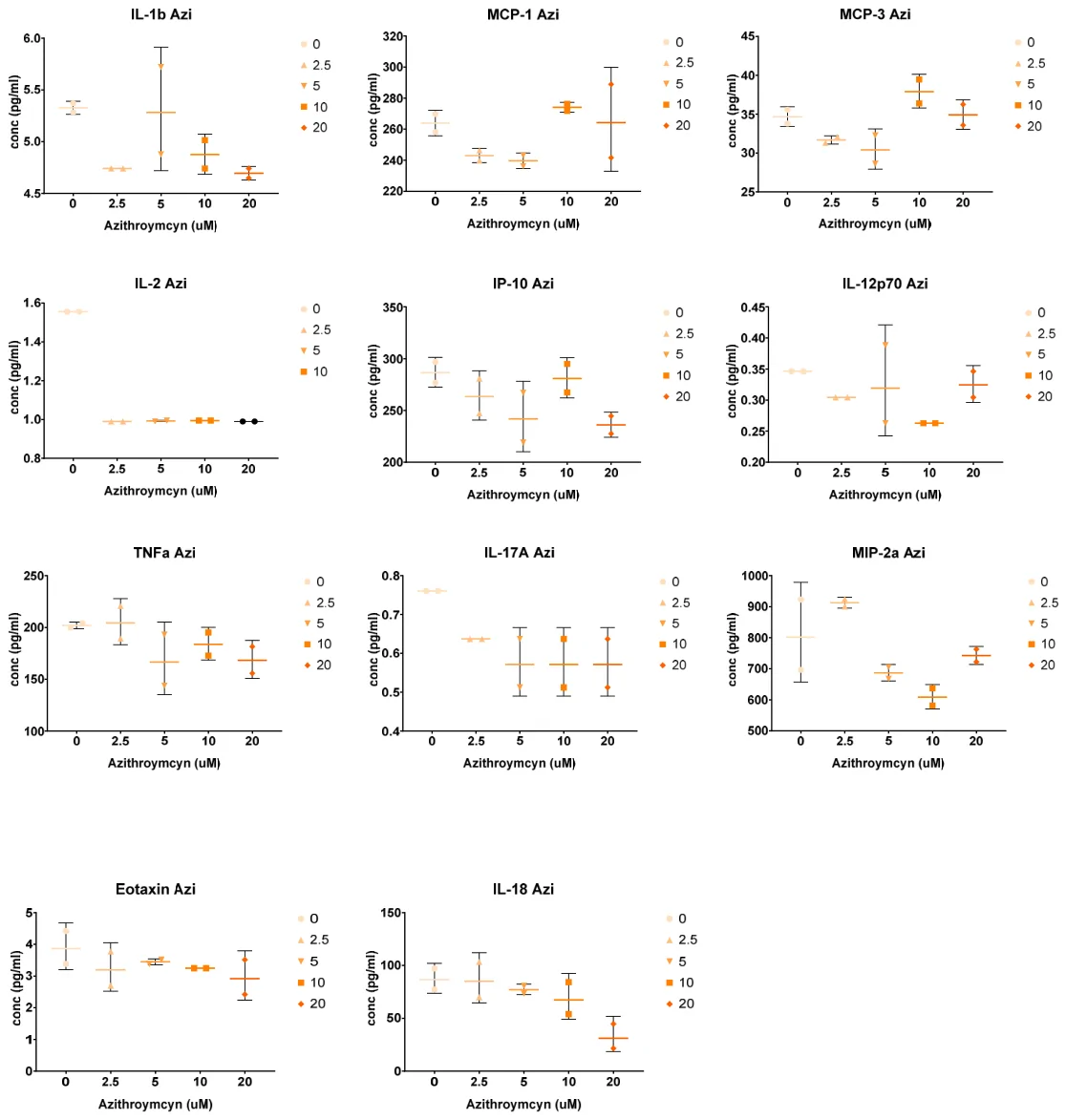
Cytokine and chemokine protein expression profiles of Nirmatrelvir and Azithromycin combination treatment against MHV infection of RAW264.7 macrophages. The *y*-axis shows the cytokine or chemokine concentration (pg/mL). The *x*-axis shows various Azithromycin (Azi) concentrations (from 0 to 20 μM) combined with 2.5 μM Nirmatrelvir. Each brown horizontal line indicates the mean protein concentration along with the standard error of the mean represented by an error bar. There was no statistical significance as determined by one-way ANOVA with Bonferroni’s multiple comparison tests against the untreated infected control. IL-4, IL-5, IL-9, IL-13, IL-22, IL-23, IFN-gamma, and GM-CSF were not detected. GRO-alpha, RANTES, MIP-1-alpha, and MIP-1-beta concentrations were beyond the upper limit of quantification.

## Data Availability

The original contributions presented in this study are included in the article. Further inquiries can be directed to the corresponding author.

## Supplementary Materials

The following supporting information can be downloaded at: https://www.mdpi.com/article/10.3390/ijms27167316/s1.

## References

[1] Zabidi N.Z., Liew H.L., Farouk I.A., Puniyamurti A., Yip A.J.W., Wijesinghe V.N., Low Z.Y., Tang J.W., Chow V.T.K., Lal S.K. (2023). Evolution of SARS-CoV-2 Variants: Implications on Immune Escape, Vaccination, Therapeutic and Diagnostic Strategies. Viruses.

[2] Coleman K.K., Tay D.J.W., Tan K.S., Ong S.W.X., Than T.S., Koh M.H., Chin Y.Q., Nasir H., Mak T.M., Chu J.J.H. (2022). Viral Load of Severe Acute Respiratory Syndrome Coronavirus 2 (SARS-CoV-2) in Respiratory Aerosols Emitted by Patients With Coronavirus Disease 2019 (COVID-19) While Breathing, Talking, and Singing. Clin. Infect. Dis..

[3] Ma K.C., Webber A., Lauring A.S., Bendall E., Papalambros L.K., Safdar B., Ginde A.A., Peltan I.D., Brown S.M., Gaglani M. (2026). Estimated Effectiveness of 2024-2025 COVID-19 Vaccination Against Severe COVID-19. JAMA Netw. Open.

[4] Wilder-Smith A., Chiew C.J., Lee V.J. (2020). Can we contain the COVID-19 outbreak with the same measures as for SARS?. Lancet Infect. Dis..

[5] von Delft A., Hall M.D., Kwong A.D., Purcell L.A., Saikatendu K.S., Schmitz U., Tallarico J.A., Lee A.A. (2023). Accelerating antiviral drug discovery: Lessons from COVID-19. Nat. Rev. Drug Discov..

[6] Ajmera H., Lakhawat S.S., Malik N., Kumar A., Bhatti J.S., Kumar V., Gogoi H., Jaswal S.K., Chandel S., Sharma P.K. (2024). Global Emergence of SARS-CoV2 Infection and Scientific Interventions to Contain its Spread. Curr. Protein Pept. Sci..

[7] Mengist H.M., Dilnessa T., Jin T. (2021). Structural Basis of Potential Inhibitors Targeting SARS-CoV-2 Main Protease. Front. Chem..

[8] Yip A.J.W., Low Z.Y., Chow V.T.K., Lal S.K. (2022). Repurposing Molnupiravir for COVID-19: The Mechanisms of Antiviral Activity. Viruses.

[9] Du S., Hu X., Li P., Xu S., Kim M., Liu X., Zhan P. (2026). Antiviral drug discovery and development: Challenges and future directions. Signal Transduct. Target. Ther..

[10] Yeo J.Q., Tan P.C., Tan C.W.Y., Lee P.S.S. (2024). COVID-19 residual symptoms and adverse drug reactions after oral antiviral therapy in the Singapore primary care setting. Ann. Acad. Med. Singap..

[11] Adhikari S., Khadka S., Dahal S., Shrestha D.B., Shahi J., Bajgain Y. (2021). Remdesivir in COVID-19 management: Availability and relevance to low- and middle-income countries. Drugs Ther. Perspect..

[12] Beigel J.H., Tomashek K.M., Dodd L.E., Mehta A.K., Zingman B.S., Kalil A.C., Hohmann E., Chu H.Y., Luetkemeyer A., Kline S. (2020). Remdesivir for the Treatment of Covid-19—Final Report. N. Engl. J. Med..

[13] Torii S., Kim K.S., Koseki J., Suzuki R., Iwanami S., Fujita Y., Jeong Y.D., Ito J., Asakura H., Nagashima M. (2023). Increased flexibility of the SARS-CoV-2 RNA-binding site causes resistance to remdesivir. PLoS Pathog..

[14] Zhou Y., Gammeltoft K.A., Ryberg L.A., Pham L.V., Tjørnelund H.D., Binderup A., Duarte Hernandez C.R., Fernandez-Antunez C., Offersgaard A., Fahnøe U. (2022). Nirmatrelvir-resistant SARS-CoV-2 variants with high fitness in an infectious cell culture system. Sci. Adv..

[15] Maitra A., Bates S., Kolvekar T., Devarajan P.V., Guzman J.D., Bhakta S. (2015). Repurposing-a ray of hope in tackling extensively drug resistance in tuberculosis. Int. J. Infect. Dis..

[16] Deeks S.G. (2011). HIV infection, inflammation, immunosenescence, and aging. Annu. Rev. Med..

[17] Riva L., Yuan S., Yin X., Martin-Sancho L., Matsunaga N., Pache L., Burgstaller-Muehlbacher S., De Jesus P.D., Teriete P., Hull M.V. (2020). Discovery of SARS-CoV-2 antiviral drugs through large-scale compound repurposing. Nature.

[18] Haldar J. (2025). Combination Therapy: A Pillar in the Fight Against Infectious Diseases. ACS Infect. Dis..

[19] Wagoner J., Herring S., Hsiang T.Y., Ianevski A., Biering S.B., Xu S., Hoffmann M., Pöhlmann S., Gale M., Aittokallio T. (2022). Combinations of Host- and Virus-Targeting Antiviral Drugs Confer Synergistic Suppression of SARS-CoV-2. Microbiol. Spectr..

[20] Tan Y.L., Tan K.S.W., Chu J.J.H., Chow V.T. (2021). Combination Treatment with Remdesivir and Ivermectin Exerts Highly Synergistic and Potent Antiviral Activity Against Murine Coronavirus Infection. Front. Cell. Infect. Microbiol..

[21] Lew R.Z.Z., Tay D.J.W., Ong J.W.X., Low J.H., Liu J., Wang Y., Chu J.J.H., Andiappan A.K., Tan K.S., Chow V.T.K. (2026). Combination of Remdesivir and Ivermectin Exerts Highly Potent and Synergistic Antiviral Activity Against Murine Coronavirus and SARS-CoV-2 Infections. Cells.

[22] Lehár J., Krueger A.S., Avery W., Heilbut A.M., Johansen L.M., Price E.R., Rickles R.J., Short G.F., Staunton J.E., Jin X. (2009). Synergistic drug combinations tend to improve therapeutically relevant selectivity. Nat. Biotechnol..

[23] Zaidi A.K., Dehgani-Mobaraki P. (2022). The mechanisms of action of ivermectin against SARS-CoV-2-an extensive review. J. Antibiot..

[24] Palacios-Rápalo S.N., Farfan-Morales C.N., Cordero-Rivera C.D., De Jesús-González L.A., Reyes-Ruiz J.M., Meraz-Ríos M.A., Del Ángel R.M. (2023). An ivermectin-atorvastatin combination impairs nuclear transport inhibiting dengue infection in vitro and in vivo. iScience.

[25] Rothan H.A., Mohamed Z., Paydar M., Rahman N.A., Yusof R. (2014). Inhibitory effect of doxycycline against dengue virus replication in vitro. Arch. Virol..

[26] Chaves Filho A.J.M., Gonçalves F., Mottin M., Andrade C.H., Fonseca S.N.S., Macedo D.S. (2021). Repurposing of Tetracyclines for COVID-19 Neurological and Neuropsychiatric Manifestations: A Valid Option to Control SARS-CoV-2-Associated Neuroinflammation?. J. Neuroimmune Pharmacol..

[27] Gyselinck I., Janssens W., Verhamme P., Vos R. (2021). Rationale for azithromycin in COVID-19: An overview of existing evidence. BMJ Open Respir. Res..

[28] Khoshnood S., Shirani M., Dalir A., Moradi M., Haddadi M.H., Sadeghifard N., Birjandi F.S., Yashmi I., Heidary M. (2022). Antiviral effects of azithromycin: A narrative review. Biomed. Pharmacother..

[29] Low Z.Y., Yip A.J.W., Lal S.K. (2022). Repositioning Ivermectin for Covid-19 treatment: Molecular mechanisms of action against SARS-CoV-2 replication. Biochim. Biophys. Acta Mol. Basis Dis..

[30] Malek A.E., Granwehr B.P., Kontoyiannis D.P. (2020). Doxycycline as a potential partner of COVID-19 therapies. IDCases.

[31] Khezri M.R., Zolbanin N.M., Ghasemnejad-Berenji M., Jafari R. (2021). Azithromycin: Immunomodulatory and antiviral properties for SARS-CoV-2 infection. Eur. J. Pharmacol..

[32] Meidaninikjeh S., Sabouni N., Marzouni H.Z., Bengar S., Khalili A., Jafari R. (2021). Monocytes and macrophages in COVID-19: Friends and foes. Life Sci..

[33] Li J., Shan R., Miller H., Filatov A., Byazrova M.G., Yang L., Liu C. (2025). The roles of macrophages and monocytes in COVID-19 Severe Respiratory Syndrome. Cell Insight.

[34] Aoki-Utsubo C., Chen M., Hotta H. (2018). Time-of-addition and Temperature-shift Assays to Determine Particular Step(s) in the Viral Life Cycle that is Blocked by Antiviral Substance(s). Bio Protoc..

[35] Ngwe Tun M.M., Luvai E., Nwe K.M., Toume K., Mizukami S., Hirayama K., Komatsu K., Morita K. (2022). Anti-SARS-CoV-2 activity of various PET-bottled Japanese green teas and tea compounds in vitro. Arch. Virol..

[36] Joyce R.P., Hu V.W., Wang J. (2022). The history, mechanism, and perspectives of nirmatrelvir (PF-07321332): An orally bioavailable main protease inhibitor used in combination with ritonavir to reduce COVID-19-related hospitalizations. Med. Chem. Res..

[37] Ng W.H., Tang P.C.H., Mahalingam S., Liu X. (2023). Repurposing of drugs targeting the cytokine storm induced by SARS-CoV-2. Br. J. Pharmacol..

[38] Faraj S.S., Jalal P.J. (2023). IL1β, IL-6, and TNF-α cytokines cooperate to modulate a complicated medical condition among COVID-19 patients: Case-control study. Ann. Med. Surg..

[39] Schultheiß C., Willscher E., Paschold L., Gottschick C., Klee B., Henkes S.S., Bosurgi L., Dutzmann J., Sedding D., Frese T. (2022). The IL-1β, IL-6, and TNF cytokine triad is associated with post-acute sequelae of COVID-19. Cell Rep. Med..

[40] Joynt G.M., Wu W.K. (2020). Understanding COVID-19: What does viral RNA load really mean?. Lancet Infect. Dis..

[41] Michalakis Y., Sofonea M.T., Alizon S., Bravo I.G. (2021). SARS-CoV-2 viral RNA levels are not ‘viral load’. Trends Microbiol..

[42] Despres H.W., Mills M.G., Shirley D.J., Schmidt M.M., Huang M.L., Roychoudhury P., Jerome K.R., Greninger A.L., Bruce E.A. (2022). Measuring infectious SARS-CoV-2 in clinical samples reveals a higher viral titer:RNA ratio for Delta and Epsilon vs. Alpha variants. Proc. Natl. Acad. Sci. USA.

[43] Dharra R., Kumar Sharma A., Datta S. (2023). Emerging aspects of cytokine storm in COVID-19: The role of proinflammatory cytokines and therapeutic prospects. Cytokine.

[44] Liu B., Li M., Zhou Z., Guan X., Xiang Y. (2020). Can we use interleukin-6 (IL-6) blockade for coronavirus disease 2019 (COVID-19)-induced cytokine release syndrome (CRS)?. J. Autoimmun..

[45] Peter A.E., Sandeep B.V., Rao B.G., Kalpana V.L. (2021). Calming the Storm: Natural Immunosuppressants as Adjuvants to Target the Cytokine Storm in COVID-19. Front. Pharmacol..

[46] Narasaraju T., Tang B.M., Herrmann M., Muller S., Chow V.T.K., Radic M. (2020). Neutrophilia and NETopathy as Key Pathologic Drivers of Progressive Lung Impairment in Patients With COVID-19. Front. Pharmacol..

[47] Mahajan S., Mahajan S., Gusain A. (2026). Interleukins in COVID-19 and SARS-CoV-2 Variants: Immunopathogenesis, Therapeutic Perspectives and Vaccine-Induced Immune Responses. Int. J. Mol. Sci..

[48] Rahmah L., Abarikwu S.O., Arero A.G., Essouma M., Jibril A.T., Fal A., Flisiak R., Makuku R., Marquez L., Mohamed K. (2022). Oral antiviral treatments for COVID-19: Opportunities and challenges. Pharmacol. Rep..

[49] McLean G., Kamil J., Lee B., Moore P., Schulz T.F., Muik A., Sahin U., Türeci Ö., Pather S. (2022). The Impact of Evolving SARS-CoV-2 Mutations and Variants on COVID-19 Vaccines. mBio.

[50] Wen W., Chen C., Tang J., Wang C., Zhou M., Cheng Y., Zhou X., Wu Q., Zhang X., Feng Z. (2022). Efficacy and safety of three new oral antiviral treatment (molnupiravir, fluvoxamine and Paxlovid) for COVID-19: A meta-analysis. Ann. Med..

[51] Esposito M.M., Turku S., Lehrfield L., Shoman A. (2023). The Impact of Human Activities on Zoonotic Infection Transmissions. Animals.

[52] Zumla A., Hui D.S., Peiris M., Perlman S. (2026). Respiratory infections due to human common cold coronaviruses, SARS-CoV, MERS-CoV, and SARS-CoV-2: Epidemiology, pathogenesis, clinical features, diagnostics, therapeutics, and vaccine landscapes. Lancet Respir. Med..

[53] Cui J., Li F., Shi Z.L. (2019). Origin and evolution of pathogenic coronaviruses. Nat. Rev. Microbiol..

[54] Sherwood M., Zhou Y., Sui Y., Wang Y., Skipp P., Kaid C., Gray J., Okamoto K., Ewing R.M. (2024). Integrated re-analysis of transcriptomic and proteomic datasets reveals potential mechanisms for Zika viral-based oncolytic therapy in neuroblastoma. F1000Research.

[55] Perrin-Cocon L., Diaz O., Jacquemin C., Barthel V., Ogire E., Ramière C., André P., Lotteau V., Vidalain P.O. (2020). The current landscape of coronavirus-host protein-protein interactions. J. Transl. Med..

[56] Iketani S., Mohri H., Culbertson B., Hong S.J., Duan Y., Luck M.I., Annavajhala M.K., Guo Y., Sheng Z., Uhlemann A.C. (2023). Multiple pathways for SARS-CoV-2 resistance to nirmatrelvir. Nature.

[57] Jeong J.H., Chokkakula S., Min S.C., Kim B.K., Choi W.S., Oh S., Yun Y.S., Kang D.H., Lee O.J., Kim E.G. (2022). Combination therapy with nirmatrelvir and molnupiravir improves the survival of SARS-CoV-2 infected mice. Antivir. Res..

[58] Akinbolade S., Coughlan D., Fairbairn R., McConkey G., Powell H., Ogunbayo D., Craig D. (2022). Combination therapies for COVID-19: An overview of the clinical trials landscape. Br. J. Clin. Pharmacol..

[59] Tang J., Wennerberg K., Aittokallio T. (2015). What is synergy? The Saariselkä agreement revisited. Front. Pharmacol..

[60] Jeffreys L.N., Pennington S.H., Duggan J., Caygill C.H., Lopeman R.C., Breen A.F., Jinks J.B., Ardrey A., Donnellan S., Patterson E.I. (2022). Remdesivir-ivermectin combination displays synergistic interaction with improved in vitro activity against SARS-CoV-2. Int. J. Antimicrob. Agents.

[61] Tan K.C., Neo J.H.Y., Tran T., Chow V.T.K. (2026). Combinations of Favipiravir with Doxycycline, Azithromycin or Ivermectin Exert Synergistic Effects Against Influenza A H3N2 Virus Replication. Pathogens.

[62] Noh H., Yoon S., Kim S.H., Kim J., Seo J.S., Kim J.J., Park I.H., Oh J., Bae J.Y., Lee G.E. (2023). Establishment of multicenter COVID-19 therapeutics preclinical test system in Republic of Korea. Pulm. Pharmacol. Ther..

[63] Cox R.M., Lieber C.M., Wolf J.D., Karimi A., Lieberman N.A.P., Sticher Z.M., Roychoudhury P., Andrews M.K., Krueger R.E., Natchus M.G. (2023). Comparing molnupiravir and nirmatrelvir/ritonavir efficacy and the effects on SARS-CoV-2 transmission in animal models. Nat. Commun..

[64] Yuan L., Tang Q., Zhu H., Guan Y., Cheng T., Xia N. (2021). SARS-CoV-2 infection and disease outcomes in non-human primate models: Advances and implications. Emerg. Microbes Infect..

