# Supplementary material for: The Combination of Nirmatrelvir and Ivermectin Exerts Strongly Synergistic Antiviral and Anti-Inflammatory Effects Against Murine Coronavirus Infection of Macrophages
Source: Int J Mol Sci. 2026 Aug 16;27(16):7316. doi: 10.3390/ijms27167316 (PMC13513154; doi:10.3390/ijms27167316)
Supplement: Supplementary file 1 [file ijms-27-07316-s001.zip › ijms-4438828-supplementary.pdf]

	**<u>Dilution of culture supernatant</u>**						
	**10^2**	**10^3**	**10^4**	**10^5**	**10^6**	**10^7**	
NMV = 0 µM<br>Untreated control<br>IVM = 0 µM	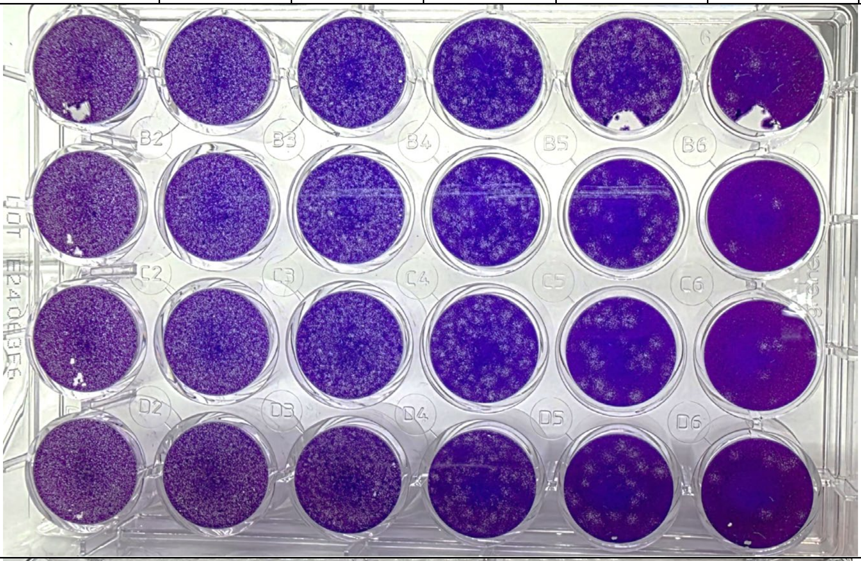						
NMV = 0 µM<br><br>IVM = 0.15 µM							
NMV = 0 µM<br><br>IVM = 0.3 µM							
NMV = 0 µM<br><br>IVM = 0.6 µM							
NMV = 0 µM<br><br>IVM = 1.2 µM	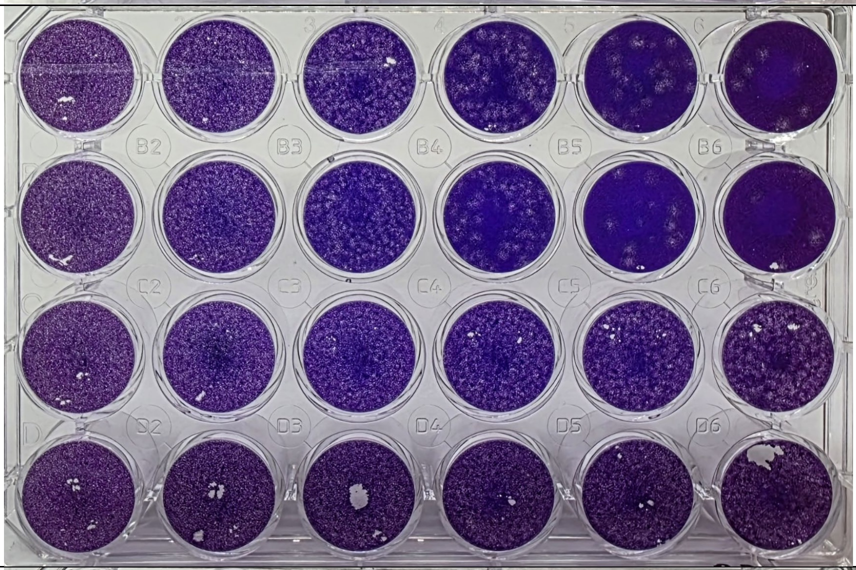						
NMV = 0 µM<br><br>IVM = 2.4 µM							
NMV = 0.625 µM<br><br>IVM = 0 µM							
NMV = 0.625 µM<br><br>IVM = 0.15 µM							
NMV = 0.625 µM<br><br>IVM = 0.3 µM	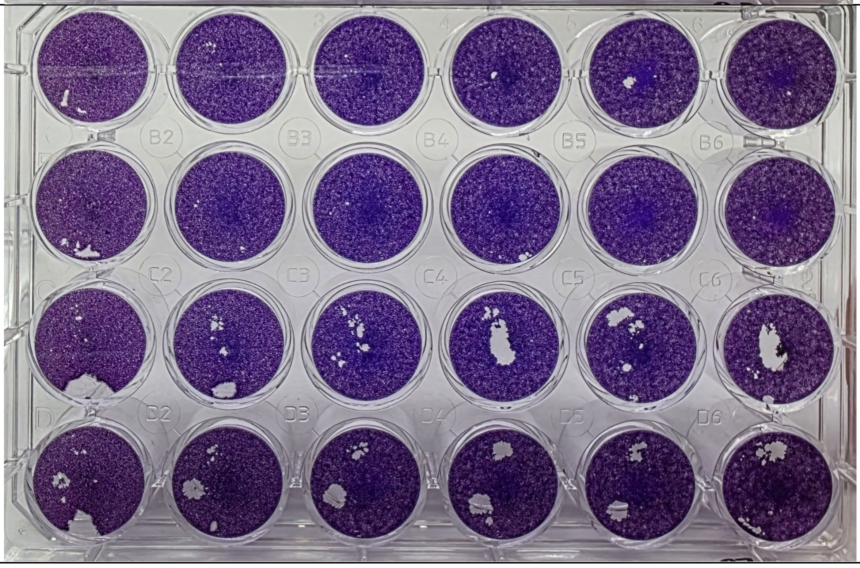						
NMV = 0.625 µM<br><br>IVM = 0.6 µM							
NMV = 0.625 µM<br><br>IVM = 1.2 µM							
NMV = 0.625 µM<br><br>IVM = 2.4 µM							

	**Dilution of culture supernatant**						
	**10^2**	**10^3**	**10^4**	**10^5**	**10^6**	**10^7**	
NMV = 1.25 µM<br>IVM = 0 µM	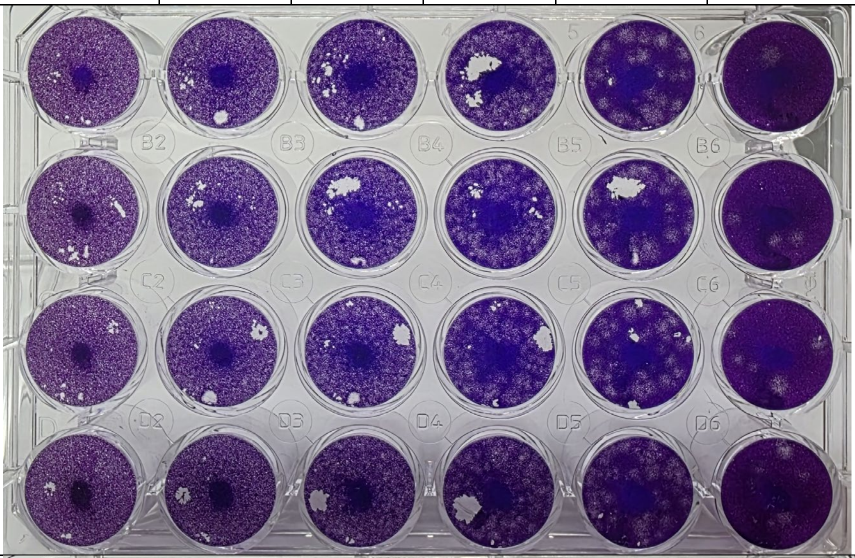						
NMV = 1.25 µM<br>IVM = 0.15 µM							
NMV = 1.25 µM<br>IVM = 0.3 µM							
NMV = 1.25 µM<br>IVM = 0.6 µM							
NMV = 1.25 µM<br>IVM = 1.2 µM	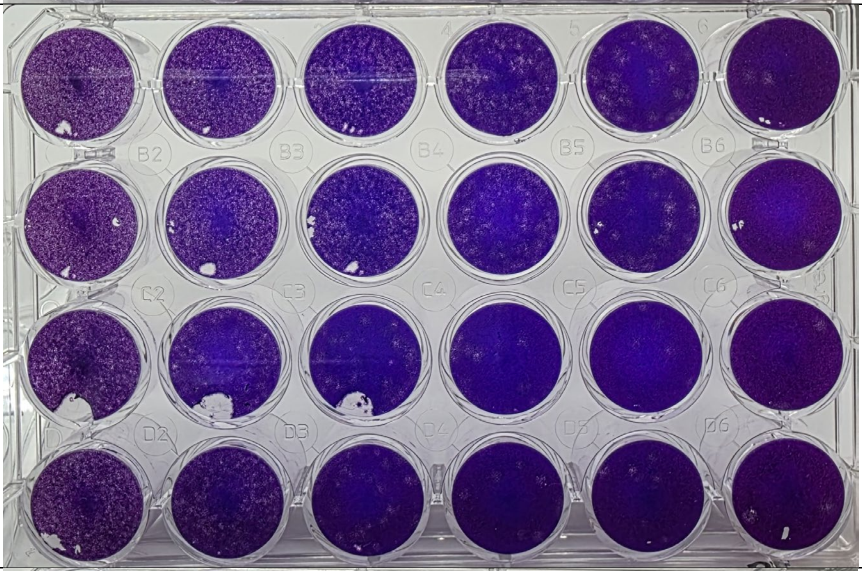						
NMV = 1.25 µM<br>IVM = 2.4 µM							
NMV = 2.5 µM<br>IVM = 0 µM							
NMV = 2.5 µM<br>IVM = 0.15 µM							
NMV = 2.5 µM<br>IVM = 0.3 µM	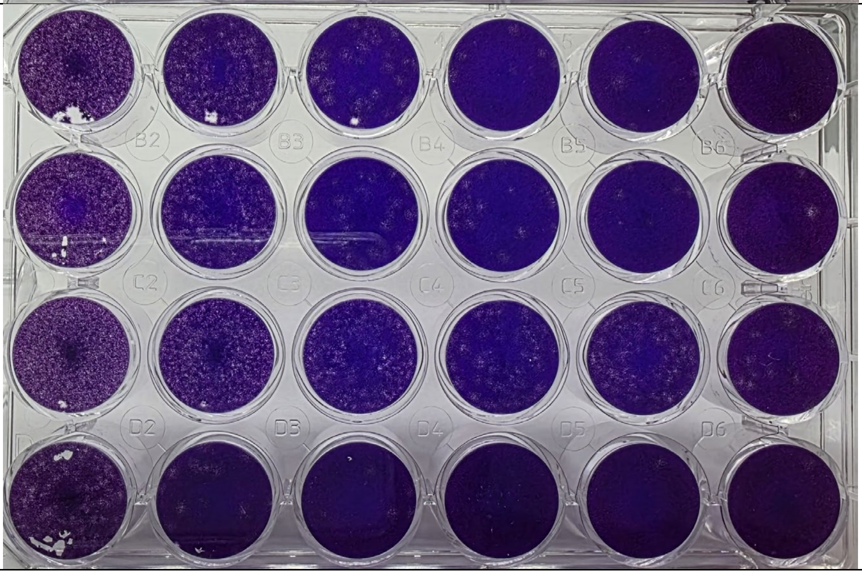						
NMV = 2.5 µM<br>IVM = 0.6 µM							
NMV = 2.5 µM<br>IVM = 1.2 µM							
NMV = 2.5 µM<br>IVM = 2.4 µM							

	Dilution of culture supernatant						
	**10^2**	**10^3**	**10^4**	**10^5**	**10^6**	**10^7**	
NMV = 5 µM<br>IVM = 0 µM	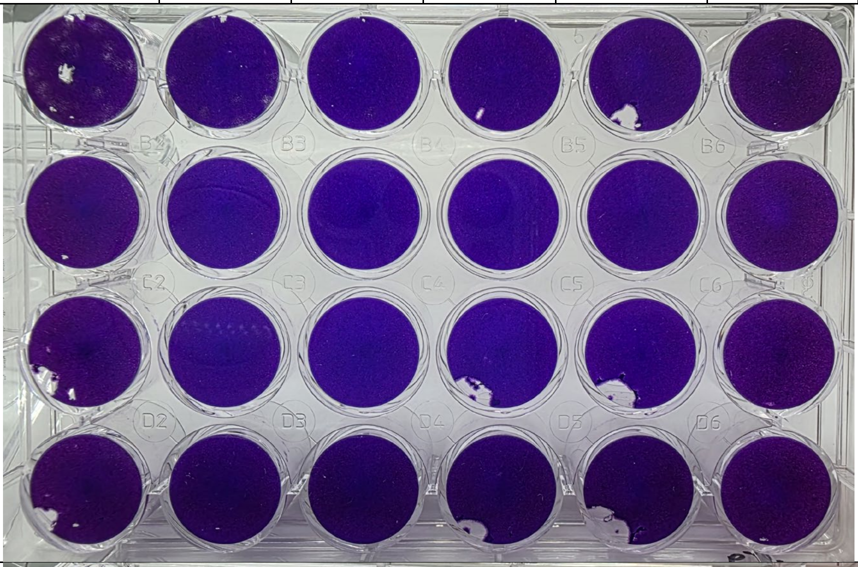						
NMV = 5 µM<br>IVM = 0.15 µM							
NMV = 5 µM<br>IVM = 0.3 µM							
NMV = 5 µM<br>IVM = 0.6 µM							
NMV = 5 µM<br>IVM = 1.2 µM	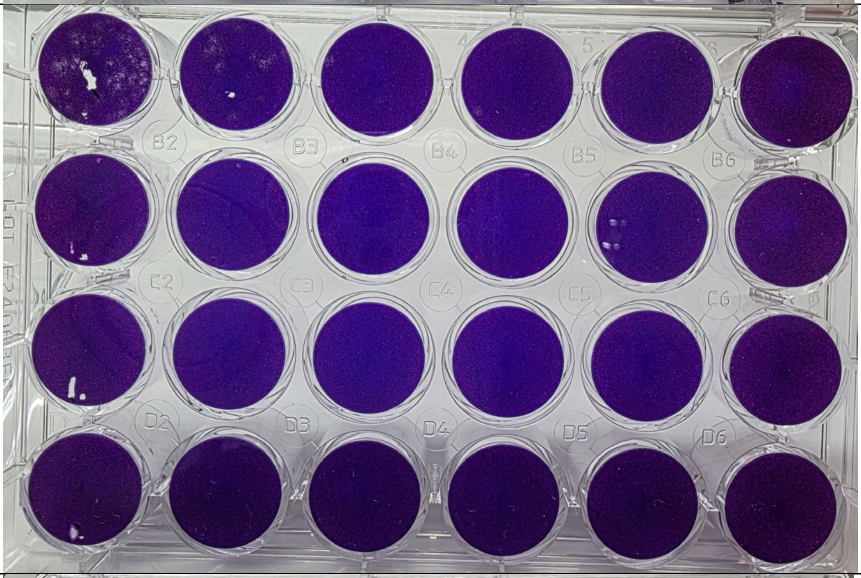						
NMV = 5 µM<br>IVM = 2.4 µM							
NMV = 10 µM<br>IVM = 0 µM							
NMV = 10 µM<br>IVM = 0.15 µM							
NMV = 10 µM<br>IVM = 0.3 µM	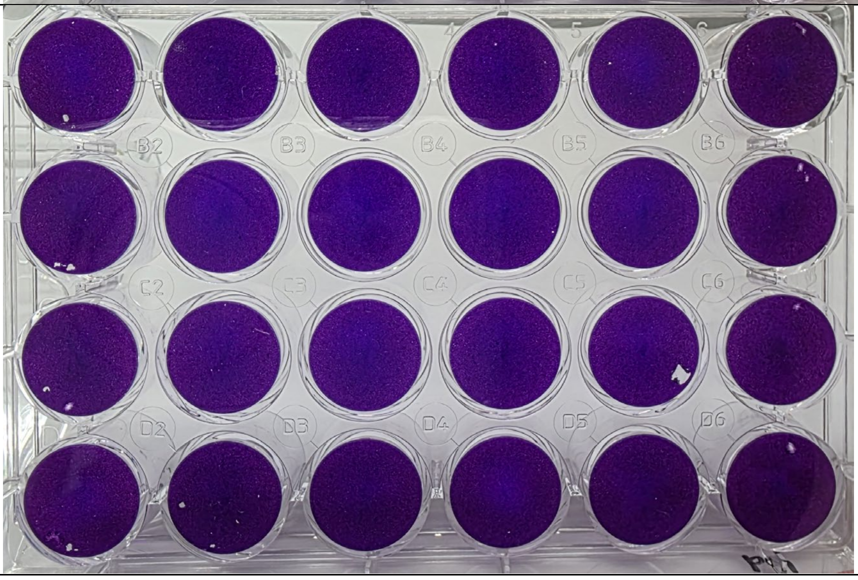						
NMV = 10 µM<br>IVM = 0.6 µM							
NMV = 10 µM<br>IVM = 1.2 µM							
NMV = 10 µM<br>IVM = 2.4 µM							

	Dilution of culture supernatant						
	10^2	10^3	10^4	10^5	10^6	10^7	
No virus infection control	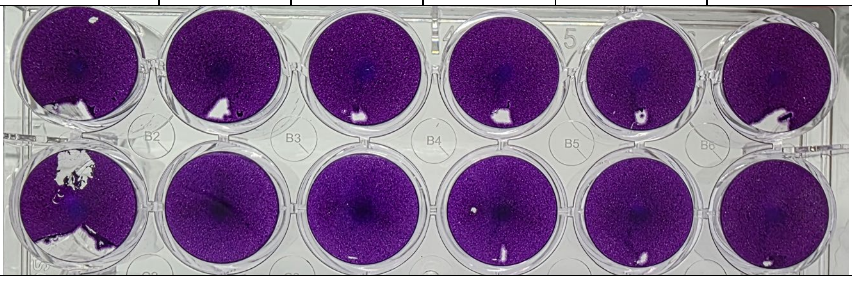						
No virus infection control							

Supplementary Figure S1. Representative virus plaque assays of culture supernatant samples of MHV-infected RAW264.7 cells treated with Nirmatrelvir (NMV) and/or Ivermectin (IVM). Ten-fold serial dilutions (from 10^2 to 10^7) of culture supernatant samples were subjected to plaque assays using H2.35 mouse liver cells. For example, no plaques were observed for treatment with 5 µM or 10 µM of Nirmatrelvir alone or combined with various concentrations of Ivermectin. Virus plaques were also absent for the uninfected control wells, whereas numerous plaques were obtained for the infected untreated control wells.
